# Emergent solidity of amorphous materials as a consequence of mechanical self-organisation

**DOI:** 10.1038/s41467-020-18663-7

**Published:** 2020-09-25

**Authors:** Hua Tong, Shiladitya Sengupta, Hajime Tanaka

**Affiliations:** 1 School of Physics and Astronomy, Shanghai Jiao Tong University, 800 Dong Chuan Road, Shanghai, 200240 China; 2 Department of Fundamental Engineering, Institute of Industrial Science, University of Tokyo, 4-6-1 Komaba, Meguro-ku, Tokyo, 153-8505 Japan; 3 Department of Physics, Indian Institute of Technology Roorkee, Roorkee, Uttarakhand 247667 India

**Keywords:** Glasses, Rheology, Glasses, Theory and computation, Structure of solids and liquids

## Abstract

Amorphous solids have peculiar properties distinct from crystals. One of the most fundamental mysteries is the emergence of solidity in such nonequilibrium, disordered state without the protection by long-range translational order. A jammed system at zero temperature, although marginally stable, has solidity stemming from the space-spanning force network, which gives rise to the long-range stress correlation. Here, we show that such nonlocal correlation already appears at the nonequilibrium glass transition upon cooling. This is surprising since we also find that the system suffers from giant anharmonic fluctuations originated from the fractal-like potential energy landscape. We reveal that it is the percolation of the force-bearing network that allows long-range stress transmission even under such circumstance. Thus, the emergent solidity of amorphous materials is a consequence of nontrivial self-organisation of the disordered mechanical architecture. Our findings point to the significance of understanding amorphous solids and nonequilibrium glass transition from a mechanical perspective.

## Abstract (web summary)

Glass materials are solid, like crystals, but lack long-range order, whilst the origin of their solidity remains elusive. Tong et al. show that the emergent solidity of glasses is induced by self-organization of percolation of force-bearing network appeared at the non-equilibrium glass transition upon cooling.

## Introduction

Amorphous solids, or glasses, are apparently rigid as a crystalline state of matter, but at the same time, disordered as a liquid state. Such a combination of rigidity and disorder remains a fundamental open question in condensed matter physics and materials science despite serious efforts over the years^1,2^. On the other hand, a crystal is in a thermodynamically and mechanically equilibrium state. Its solidity is maintained by the long-range translational order, which may survive even under thermal noise as long as the long-range order persists. A glass is, on the other hand, in a thermodynamically nonequilibrium state without long-range translational order. It is, therefore, puzzling what can be the general principle of organisation for amorphous solids. Recently, a step forward towards the solution may have emerged: the critical consequence of mechanical stability in the inherent state (IS) of the glass, i.e., the zero-temperature state in mechanical equilibrium in the absence of the thermal fluctuations, has been shown in the form of long-range static stress correlation, which decays as a power-law of 1/*r*^*d*^ in *d* dimensions^3–7^. This finding provides a theoretical basis for the earlier studies of stress or strain correlations in both simulations^3,4,8–11^ and experiments^10–14^. More importantly, the existence of long-range stress correlations may serve as the foundation to understand the emergence of rigidity^15–17^ of amorphous solids and their unique properties observed in experiments^1,2,11^, e.g., the excess of sound attenuation compared with the Rayleigh prediction^18^, but only if its relevance at finite temperatures can be established.

The expectation that the low-temperature properties of solids necessarily reflect the IS at zero temperature largely relies on the solid-state physics of crystals^19^. This view has been largely taken for granted and widely applied to the studies of amorphous solids and glass transition, e.g., in the celebrated potential energy landscape (PEL) description for glassy systems^20,21^. However, its validity is obscure because of the lack of long-range translational order in amorphous materials. Indeed, the challenge comes from two lines of recent researches on glasses. The first focuses on the zero-temperature amorphous solid itself. Based on the vibrational mode analysis^22–24^, the study of plastic behaviours in strained amorphous solids^25,26^, and by means of variational arguments and effective medium theory^27,28^, it is revealed that amorphous solids are marginally stable^26–30^, with soft modes at vanishingly small frequencies and plastic responses at vanishingly small deformations. We note that these features were observed for various interaction potentials (repulsive and attractive)^22,23^ and for 2, 3 and 4 dimensions^24^. Another research stream focuses on the finite-temperature hard-sphere glasses from mean-field theories^31,32^. Marginal stability of amorphous solids is predicted to root in the Gardner transition from a normal glass phase into a marginally stable one^31,32^, which leads to hierarchical dynamics at low temperatures much more complex than simple vibrations expected from the original PEL picture^33^. Consequently, the marginal stability intrinsically cuts off the direct link between properties of amorphous solids at zero and finite temperatures^34,35^, although the concept of marginal stability may not be universally valid in a strict sense for all amorphous solids^36^. Thus, we may conclude that there is actually no solid base to deduce the properties of thermal amorphous solids directly from the IS. Thus, whether the long-range stress correlation exists in thermal amorphous solids remains a fundamental open question; and if yes, what is the underlying physical mechanism?

The emergence of long-range stress correlations in amorphous solids has also been discussed as a consequence of the ideal liquid-to-glass transition where the relaxation time *τ* diverges, by mode-coupling theories^9,10,37^, mean-field replica approaches^17^ and the fluctuation–dissipation theorem^38^. However, it is argued that the long-range character of stress correlations is established through momentum propagation/conservation^9,10,38^, which is therefore dynamical in nature, distinct from the above static approach for the correlations^4–7^. The relevance of these mean-field predictions in experimental glasses^10–14^ and the relations between these dynamical approaches and the above-mentioned static ones remain to be examined. Concerning these questions, it is of crucial importance to recognise that amorphous solids are intrinsically out-of-equilibrium states of matter, the properties of which depend on the history of preparations^2^. The critical question is, therefore, concerned with the emergence of long-range stress correlations across the protocol-dependent nonequilibrium glass transition.

To this end, we systematically study the spatial correlation of the shear-stress field and check the validity of harmonic approximation in a temperature range in which the system transforms from a supercooled liquid to a low-temperature solid-state. We induce the nonequilibrium glass transition by continuous cooling with a constant rate of *γ*, similarly to experiments. Strikingly, we find the emergence of long-range stress correlation below a cooling-rate dependent glass transition temperature *T*_g_(*γ*), at which the system falls out of equilibrium and gets trapped in a metastable glass state. However, the observed long-range stress correlation in thermal amorphous solids cannot be explained by straightforwardly applying simple harmonic approximations to that in the inherent state at zero temperature. This is because the correlation is established under giant anharmonicity, which is revealed in terms of the breakdown of force balance and the substantial deviation from a harmonic energy expansion, and more importantly, the fractal-like structure of PEL. This fact indicates that the thermal fluctuations are non-perturbative even at very low temperatures (*T* ≲ *T*_g_/10), i.e., for most of the physically relevant temperature region of real glasses. Therefore, the nature of the long-range correlation in finite-temperature amorphous solids is intrinsically different from that at zero temperature. Instead, we show that the long-range stress correlation in thermal amorphous solids emerges as a consequence of the effective mechanical equilibrium maintained by a subset of particles, or by a fraction of degrees of freedom in the high-dimensional configuration space. We identify the presence of force-bearing networks which percolate across the nonequilibrium (laboratory) glass transition *T*_g_(*γ*) upon cooling and demonstrate its general relation with the long-range stress correlation in the large system-size limit. Thus, our study crucially establishes the emergence of long-range stress correlations under giant anharmonic effects at finite temperatures, shedding new light on the very nature of amorphous solids from the mechanical perspective.

## Results

To address the questions described above, we employ binary particle mixtures interacting with finite-range repulsive potentials, which is widely used in the context of both jamming transition at zero temperature and glass transition (see ‘Methods’ on the details of this system and additional systems which are employed to confirm the generality of our findings). The simple nature of the potential allows us to study very large ensembles to precisely access the emergence of long-range stress correlations even under significant thermal fluctuations.

### Emergence of the long-range stress correlation

**Fig. 1:**
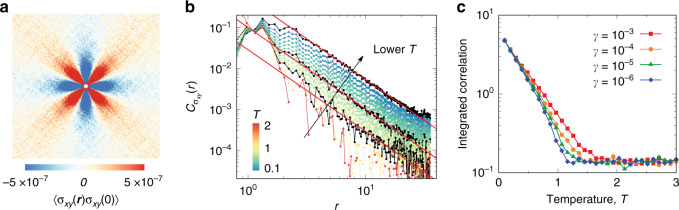
Long-range stress correlation at finite temperatures. **a** Spatial correlation of shear stress *σ*_*xy*_ at *T* = 0.1. **b** Angle-averaged shear-stress correlation ${C}_{{\sigma }_{xy}}(r)$ at temperatures covering both liquid and solid regimes. The ensemble of configurations is generated by a constant cooling rate *γ* = 10^−5^. The data colour changes from red to blue with a decrease in *T*. Three sets of data are highlighted in black colour, which, from top to bottom, correspond to *T* = 0, the hypothesized ideal glass transition temperature *T*_0_ ≈ 0.63, and slightly below the glass transition temperature *T*_g_ ≈ 1.3. The red solid lines are fits to the power-law function of 1/*r*^2^, indicating the emergence of the long-range correlation below *T*_g_. **c** Temperature dependence of integrated shear-stress correlations for different cooling rates *γ*. The *γ*-dependent non-equilibrium glass transition is signalled by the onset of the growth of shear stress correlation (see Fig. 5 below for the corresponding analysis of shear modulus). See text for detailed definitions.

We start with a 2D plot of the shear-stress correlation 〈*σ*_*xy*_(**r**)*σ*_*xy*_(0)〉 in low-temperature amorphous solids to give an overview of its spatial structure, as shown in Fig. 1a. Here *σ*_*xy*_(**r**) is the particle-level shear stress defined in ‘Methods’. Alternatively, the coarse-grained stress field can be used, which would produce essentially the same results^3^. Consistent with the zero-temperature analyses^3,4,6^, we see a long-range four-fold anisotropic feature, which suggests the existence of long-range stress correlation at finite temperatures. To quantitatively follow the evolution of stress correlations with the change of temperature, we perform an angular average of the 2D correlation function while taking care of the anisotropy ${C}_{{\sigma }_{xy}}(r)=-\frac{1}{2\pi }\mathop{\int}\nolimits_{0}^{2\pi }d\theta \langle {\sigma }_{xy}({\bf{r}}){\sigma }_{xy}(0)\rangle \cos (4\theta )/\langle {\sigma }_{xy}^{2}\rangle$, where *θ* is the angle between **r** and *x*-axis. The behaviour of ${C}_{{\sigma }_{xy}}(r)$ in the temperature range from the liquid regime too deep in the solid phase is shown in Fig. 1b. We see that ${C}_{{\sigma }_{xy}}(r)$ decays extremely fast in the liquid regime and a power-law feature develops, only when the system falls out of equilibrium, or becomes glass. Data sets for three characteristic temperatures (from top to bottom, *T* = 0 corresponding to IS, *T*_0_ the hypothesised ideal glass transition temperature from the VFT fitting of the structural relaxation time, and a temperature slightly below the glass transition temperature *T*_g_) are highlighted in black colour together with fittings to the power-law of 1/*r*^2^. This result is intuitively reasonable but at the same time surprising because the marginal stability of zero-temperature amorphous solids suggests that intrinsically different features might be observed. Here we note that to resolve the nontrivial (weak) stress correlation close to *T*_g_, a very large ensemble of independent configurations (~2000 trajectories) are necessary for the calculations. Details on the convergence of numerics and the finite-size effects are given in Supplementary Figs. 4 and 5.

In practice, amorphous solids, or glasses, are formed at the glass-transition temperature *T*_g_ upon continuous cooling (or compression), and their properties crucially depend on the protocol. To access such nonequilibrium effects on the stress correlations, we study the integrated correlation that characterises the strength of the correlation ${C}_{{\rm{int}}}=\mathop{\int}\nolimits_{{L}_{\min }}^{L/2}dr2\pi r| {C}_{{\sigma }_{xy}}(r)|$, where *L* is the linear size of the system, and we set ${L}_{\min }=4$ to focus on the far-field behaviour (so in principle, large ${L}_{\min }$ is preferred). Therefore, by definition, *C*_int_ vanishes for correlations of a shorter length scale than ${L}_{\min }$. We note that the results are insensitive to the choice of ${L}_{\min }$. It is also worth noting that, by definition, *C*_int_ diverges for a power-law correlation ${C}_{{\sigma }_{xy}}=\kappa {r}^{-2}$ in the limit of *L* → *∞*. Therefore, *C*_int_ should be regarded as a parameter characterising the relative difference between different state points; and hence it should be considered as the relative strength rather than the absolute strength of shear-stress correlations. In principle, *C*_int_ vanishes for short-range correlations and reflects the overall amplitude *κ* for the long-range power-law correlation. The emergence of nontrivial shear-stress correlations across the cooling-rate dependent glass transition is shown in Fig. 1c, signalled by the sudden growth of correlation with decreasing temperature (the noise level at high temperatures is due to numerical errors). This result proves that the long-range stress correlation emerges at the glass transition upon cooling, i.e., when the system falls out of equilibrium and becomes trapped in the metastable glass state. Considering that the cooling-rate dependent glass transition is nonequilibrium in nature, the long-range stress correlation at finite temperatures intrinsically inherits such nonequilibrium character.

### Giant anharmonic effects in thermal amorphous solids

**Fig. 2:**
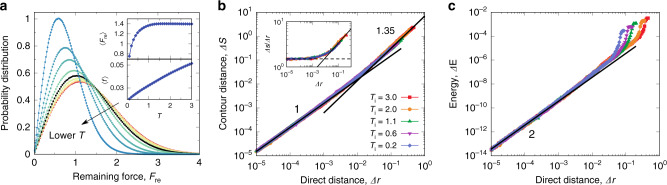
Giant anharmonic effects in thermal amorphous solids. **a** Temperature evolution of the probability distributions of rescaled remaining force ${F}_{{\rm{re}}}$ on each particle. ${F}_{{\rm{re}}}$ is defined as the ratio of the remaining force to the average strength of contacting forces, which measures the degree of deviation from the mechanical equilibrium of each particle. Here only particles with at least three contacting neighbours (the minimal requirement for local stability) are counted. We confirm that the others have little contribution to the stable force network. The same colour bar as in Fig. 1b is used. The data set corresponding to the hypothesised ideal glass transition temperature *T*_0_ ≈ 0.63 is highlighted in black colour as a reference. The lowest temperature is *T* = 0.1 in this plot. Inset: Temperature dependence of the average rescaled remaining force $\langle {F}_{{\rm{re}}}\rangle$ (top) and the globally averaged strength of force bonds 〈*f*〉 (bottom). Note that $\langle {F}_{{\rm{re}}}\rangle$ remains in the order of 1 even deep in the solid phase. **b**, **c** Characterisation of the potential energy landscape. Finite-temperature configurations, which correspond to the top-right end of each curve, are relaxed to the inherent states (IS) using energy minimisation (EM) methods. **b** Relationship between contour distance Δ*s* and direct distance Δ*r* with respect to IS during EM for different initial temperatures *T*_i_. Power-law fits are labelled by their corresponding exponent. We can see a crossover from a linear relation Δ*s* ~ Δ*r* close to IS to a fractal one Δ*s* ~ Δ*r*^1.35^ away from IS. Inset: Δ*s*/Δ*r* verse Δ*r* replotted using the data from the main panel. The crossover from Δ*s* ~ Δ*r* (dashed line) to Δ*s* ~ Δ*r*^1.35^ (solid line) is more evident in this plot. **c** Relationship between energy Δ*E* with respect to IS and Δ*r*. The solid black lines indicate the quadratic (harmonic) relation Δ*E* ~ Δ*r*^2^. Here the ensemble of configurations generated by a cooling rate *γ* = 10^−5^ is used for analyses, but the results are insensitive to the choices of *γ*. All these analyses indicate that the thermal systems in physically relevant temperatures (e.g., *T*_i_ = 0.2 ~ 3.0 in (**b**) and (**c**) are completely out of the harmonic regime).

To directly examine whether the observed finite-temperature long-range stress correlation derives from its existence at zero temperature, which is rooted in the condition of mechanical equilibrium, we calculate the remaining unbalanced force on each particle. Specifically, for particle *i*, the rescaled remaining force is defined as ${F}_{{\rm{re}}}^{i}=| {\sum }_{j}{{\bf{f}}}_{ij}| /\langle f\rangle$, where **f**_*ij*_ is the force acting on particle *i* from *j*, and 〈*f*〉 is the globally averaged strength of force bonds. We show the probability distribution of the remaining force in Fig. 2a. Obviously, ${F}_{{\rm{re}}}$ remains in the order of 1 even deep in the solid phase, indicating the serious breakdown of force balance. This result strongly suggests that the simple expectation that the long-range stress correlation in thermal amorphous solids can be explained by a harmonic approximation to the zero-temperature mechanical equilibrium is not valid, unlike in the case of crystals.

We further characterize the giant anharmonic effects from the perspective of PEL. Starting from the finite temperature configurations, we remove the thermal noise and relax the system down to the nearest energy minimum, namely IS in the basin of attraction^20,21^, using the fast inertial relaxation engine (FIRE) algorithm^39^ (we confirm that the results are robust for different parameters inside FIRE and another steepest-descent algorithm^40^). We study the structure of the paths taken by the system during the downhill motion in the *dN*-dimensional configuration space. In particular, we measure three quantities of interest: (1) the direct (end-to-end) distance from a state point in the path to the corresponding IS $\Delta r=\sqrt{{\sum }_{i}| {{\bf{r}}}_{i}-{{\bf{r}}}_{i}^{{\rm{IS}}}{| }^{2}/N}$, where **r**_*i*_ and ${{\bf{r}}}_{i}^{{\rm{IS}}}$ are the positions of particle *i* in the path and in IS, respectively; (2) the corresponding contour distance $\Delta s={\sum }_{l}\Delta {R}_{l}/\sqrt{N}$ with $\Delta {R}_{l}=\sqrt{{\sum }_{i}| {{\bf{r}}}_{i}(l)-{{\bf{r}}}_{i}(l-1){| }^{2}}$. Here, *l* indicates the number of FIRE steps away from IS labelled *l* = 0, the summation goes from 1 to the state point in the path, and **r**_*i*_(*l*) denotes the position of particle *i* in step *l*, and *l* = 0 indicating IS; and (3) the potential energy difference with respect to IS Δ*E* = (*U* − *U*^IS^)/*N*, with *U* and *U*^IS^ being the total potential energy in the path and in IS, respectively. We first examine the relation between Δ*s* and Δ*r*, which reflects the geometry of PEL, as shown in Fig. 2b. A linear relation Δ*s* ~ Δ*r* is found close to IS (small Δ*r* side), suggesting a simple downhill path and hence a simple geometry of PEL. Interestingly, at a larger distance from IS, the curve crossovers to a power-law function $\Delta s \sim \Delta {r}^{{d}_{{\rm{PEL}}}}$ with *d*_PEL_ = 1.35, suggesting a fractal-like geometry of the downhill path^41^. Therefore, in this regime, the morphology of PEL is complex and shows a certain kind of self-similarity when inspected with respect to IS. Within numerical precision, we find the fractal dimension *d*_PEL_ to be invariant when probed from different initial temperatures. Importantly, we have confirmed the same behaviour and scaling exponent in 3D harmonic and 2D Lennard-Jones systems (see Supplementary Fig. 12), strongly suggesting its relevance across different amorphous materials. This result points to an intriguing universal fractal-like structure of PEL in the physically relevant regions of the configuration space (the starting points of the curves in the large Δ*r* end). We note that similar observations have been made for the trajectory of evolving bubble packings^42^, which may also be related to the fractal free-energy landscape predicted in mean-field hard-sphere glasses^31^. Such a complex structure of PEL definitively rules out the effectiveness of harmonic approximations to the understanding of the thermal amorphous solids based on their IS. This finding calls for a reconsideration of the effectiveness of the PEL framework for a quantitative understanding of amorphous solids, not to mention the supercooled liquids.

Moreover, we show the relation between Δ*E* and Δ*r* in Fig. 2c to check the energy expansion. As expected, the quadratic relation Δ*E* ~ Δ*r*^2^ is observed close to IS, which suggests that a harmonic description is nevertheless practicable at low enough temperatures, in line with previous studies^43,44^. A strong deviation from the harmonic approximation is observed at approximately the same Δ*r* where the crossover into a fractal-like PEL happens. This result further confirms the giant anharmonic effects in thermal amorphous solids in the temperature range studied (see Supplementary Fig. 6 for further analysis on pressure). Conservatively, we estimate the crossover at Δ*r* ≈ 10^−2^ (see Fig. 2b), which corresponds to an energy scale Δ*E* ≲ 10^−6^ (see Fig. 2c) and therefore a temperature scale 10^−3^ (we note that temperature is in the unit of 10^−3^*ϵ*/*k*_B_). This suggests that the harmonic approximations are applicable only below a characteristic temperature <10^−3^*T*_g_ (see Fig. 1c for *T*_g_), which is out of physical relevance in our thermal systems, or in usual experimental conditions^10–14^. Therefore, in principle, there is no solid base to deduce the properties of thermal amorphous solids from the IS before we have a deep understanding of the fractal-like PEL.

### Percolation of force-bearing network

The coexistence of the long-range stress correlation and giant anharmonicity appears paradoxical and counterintuitive. Without the condition of mechanical equilibrium^4,5,7^, what can be the underlying mechanism leading to the emergence of long-range stress correlation? From the viewpoint of PEL, a possible physical picture is that, although mechanical equilibrium is not valid for all degrees of freedom unlike in the zero-temperature case, it is nevertheless adequately satisfied in a sufficient fraction of them. Such a condition, which we call ‘partial mechanical equilibrium’, may provide constraints necessary for the long-range stress correlation to emerge in thermal amorphous solids. However, due to the complexity of PEL, it is unfeasible to directly resolve the ‘partial mechanical equilibrium’ in the high-dimensional configuration space. Instead, thus, we seek an underlying mechanism leading to such a scenario in the real space.

**Fig. 3:**
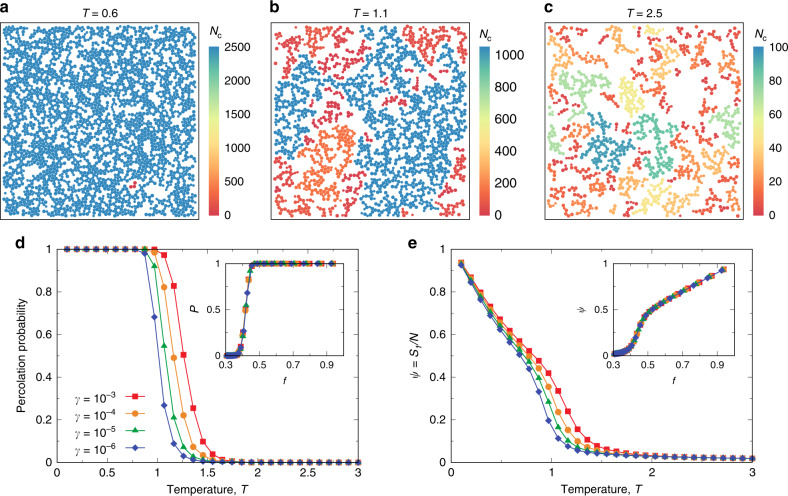
Percolation of force-bearing particles across the nonequilibrium glass transition. **a**–**c** Spatial distribution of force-bearing particles at different temperatures in the solid phase (**a**), at the critical temperature (**b**), and in the liquid regime (**c**). Configurations are from a cooling rate of *γ* = 10^−4^. Particles in the same force-bearing network are clustered and coloured according to the cluster size *N*_c_. Clusters with <3 particles are not shown for clarity. **d** Temperature dependence of percolation probability *P* for different *γ*. The *γ*-dependent nonequilibrium glass transition is signalled by the percolation of force-bearing particles. Inset: Collapse of *P* from different *γ* when replotted as a function of the fraction of force-bearing particles *f*. **e** Temperature dependence of the relative size of the largest force-bearing cluster *ψ* = *s*_1_/*N* for different *γ*, with *s*_1_ being the number of particles involved in the largest cluster. A strong similarity can be seen in comparison with the integrated stress-stress correlation in Fig. 1c. Inset: Collapse of *ψ* from different *γ* when replotted as a function of *f*. Therefore, while the percolation of force-bearing particles is controlled by *T* with different functional forms depending on *γ*, it is uniquely determined by *f*.

**Fig. 4:**
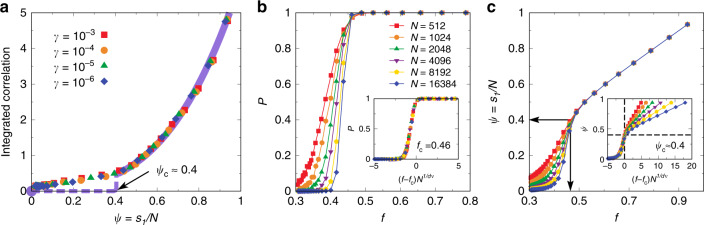
Percolation of force-bearing particles gives rise to long-range stress correlation. **a** Corresponding to Fig. 1c, the integrated shear-stress correlations for different cooling rates *γ* are plotted as a function of the relative size of the largest force-bearing network *ψ* = *s*_1_/*N*, with *N* = 4096. The nice collapse of the data at different *γ* suggests that the shear-stress correlation, more specifically, its far-field behaviour, is uniquely controlled by the largest force-bearing network. Note that many high-temperature data points of liquids have rather low but finite values of both the integrated correlation and *ψ*, and the nonequilibrium glass transition is signalled by the rapid growth of both of them. However, the finite-size scaling of *ψ* (see **b** and **c**) reveals that the non-zero values of both the integrated shear-stress correlation and *ψ* below the percolation threshold (left-lower side) actually originate from finite-size effects. Therefore, in the large system-size limit, the filled circle at the origin should describe all liquid states. Then, the dashed line indicates the abrupt emergence of percolated force-bearing network and long-range stress correlations across the nonequilibrium glass transition, and the solid line illustrates the general relation between them for a further decrease of temperature. **b** Percolation probability *P* as a function of the fraction of force-bearing particles *f* for different system-sizes *N* at *γ* = 10^−4^. Inset: Scaling collapse of *P* from different system sizes. Here *d* = 2 is the spatial dimension, *ν* = 4/3 is the scaling exponent, and *f*_c_ = 0.46 is the critical occupation fraction. **c** Corresponding to (**b**), the relative size of the largest cluster *ψ* = *s*_1_/*N* as a function of *f* for different system sizes. The vertical and horizontal arrows indicate *f*_c_ = 0.46 and the corresponding *ψ*_c_, respectively. Inset: Scaling collapse of *ψ* from different system sizes below the percolation transition, using the same *f*_c_ and *ν* as the inset of (**b**). The horizontal dashed line indicates *ψ*_c_ ≈ 0.4 at the percolation threshold *f*_c_. Taken together, both (**b**) and (**c**) indicate that, when *N* → *∞*, an abrupt formation of system-spanning force-bearing network takes place across the nonequilibrium glass transition.

In analogy with the force chains that are responsible for the rigidity of granular packings^45,46^, particles involved in a strong force network are expected to be responsible for the rigidity of thermal amorphous solids. These force-bearing particles (see ‘Methods’) may contribute to the effective mechanical equilibrium and give rise to the emergence of the long-range stress correlation. Indeed, we find that more and more force-bearing particles emerge with decreasing temperature and form a space-spanning network across the nonequilibrium glass transition, as illustrated in Fig. 3a–c. Quantitatively, we characterise the clustering of force-bearing particles and identify the percolation transition, i.e., the formation of a system-spanning force-bearing network, upon cooling with a constant rate of *γ*. Figure 3d shows the temperature dependence of the percolation probability (see ‘Methods’) for several different cooling rates. We see that the percolation of force-bearing particles takes place at a *γ*-dependent glass transition temperature, in good agreement with the emergence of the long-range shear-stress correlation upon cooling (see Fig. 1c). Furthermore, of particular importance is the largest force-bearing cluster, which controls the stress correlation at long distances. In Fig. 3e, we show the temperature dependence of the relative size of the largest force-bearing cluster *ψ* = *s*_1_/*N* for several different *γ*, with *s*_1_ being the number of particles involved in the largest cluster. We can see a close similarity between Fig. 3e and Fig. 1c, strongly suggesting a direct causal relationship between the force-bearing space-spanning network and the long-range stress correlation. Before proceeding to explore such an intimate relation (which we detail in Fig. 4a in the following section), we note that while the percolation of force-bearing particles appears to be controlled by temperature with different functional forms depending on *γ* (see the main panels of Fig. 3d, e), it is after all uniquely determined by *f*, the fraction of force-bearing particles in the system (see the data collapse in the inset of Fig. 3d). The one-to-one correspondence of *ψ* with *f* shown in the inset of Fig. 3e further indicates that the space-spanning force-bearing network is responsible for the emergence of rigidity in thermal amorphous solids upon cooling. This result crucially establishes the underlying physical mechanism for the long-range spatial correlations observed in experiments^10–14^.

### Relation between stress correlation and force network

Motivated by the above result, we plot the integrated shear-stress correlations (see Fig. 1c) as a function of the relative size of the largest force-bearing network *ψ* in Fig. 4a. An excellent collapse of the data for different *γ* is obtained. This result decisively proves that the shear-stress correlation, more specifically, its far-field behaviour, is uniquely controlled by the largest force-bearing network. In other words, the ‘partial mechanical equilibrium’ realised in the percolating force-bearing network gives rise to the long-range stress correlation in thermal amorphous solids. Since numerical simulations are inevitably limited to finite system sizes, when we talk about long-range correlations, an important question is how the observations are relevant in the large system-size limit. To address this fundamental problem, we employ a finite-size scaling analysis. As shown in Fig. 4b, the transition of percolation probability *P* from 0 to 1 as a function of the fraction of force-bearing particles *f* becomes steeper with increasing the system size *N*. Scaling collapse of *P* is realized in the inset of Fig. 4b, indicating a well-defined percolation transition at *f*_c_ = 0.46 in the large system-size limit. Correspondingly, the relative size of the largest force-bearing network *ψ* as a function of *f* is shown in Fig. 4c. The first observation is that the growth of *ψ* with increasing *f* becomes more abrupt with increasing *N*. In particular, the initiation of the growth of *ψ* approaches *f*_c_ with increasing *N*, since in the limit of *N* → *∞* a system-spanning cluster with a finite value of *ψ* = *s*_1_/*N* is impossible before the percolation transition. The scaling collapse of *ψ* below *f*_c_ shown in the inset of Fig. 4c confirms that a system-spanning force-bearing network with *ψ*_c_ ≈ 0.4 emerges abruptly at *f*_c_ as *N* → *∞*. After the percolation transition, we observe that *ψ* is controlled by *f* without noticeable finite-size effects. Therefore, there is a well-defined percolation transition of the force-bearing network at *f*_c_ = 0.46 in the large system-size limit, which corresponds to the nonequilibrium glass transition. Taking into account the finite-size scaling of the percolation transition, we deduce the behaviour of long-range stress correlations in the large system-size limit in Fig. 4a. At high temperatures, i.e., before percolation, *ψ* stays at zero and the stress correlation is short-ranged. Therefore, the filled circle at the origin describes all liquid states. So the non-zero integrated shear-stress correlation and *ψ* below the percolation threshold (left-lower side of finite-size data points) actually originate from the finite-size effects. Across the nonequilibrium glass transition upon cooling, the abrupt simultaneous emergence of percolated force-bearing network and long-range stress correlations take place, which is indicated by the dashed line. In the low-temperature solid phase, the stress correlation is long-ranged, and its relation with the percolated force-bearing network is illustrated by the solid line. With more particles involved in the percolated force-bearing network, the overall amplitude of the long-range stress correlation in thermal amorphous solids increases with lowering the temperature. We note that in a jammed system at zero temperature, almost all the particles participate in the force-bearing network besides rattlers.

In the context of percolation transition, we find that the percolation of force-bearing network is characterized by critical exponents: *ν* = 4/3 for spatial correlation (see Fig. 4b, c), *τ* = 1.85 for cluster-size distribution, and a fractal dimension *d*_f_ = 1.98 of the percolating cluster (see Supplementary Fig. 8). In comparison, the standard connectivity percolation is characterized by *ν* = 4/3, *τ* = 187/91 and *d*_f_ = 91/48^47^; and the standard rigidity percolation by *ν* = 1.21 and *d*_f_ = 1.86^48,49^. Therefore, the critical exponents we find in the percolation of force-bearing network appear to differ from those of standard connectivity and rigidity percolation, which indicates different universality classes that they belong to. It is known that the critical exponents of random percolation are very robust against the change of model details^47,50^. For example, they do not depend on the short-range structure of the lattice (e.g., square or triangular) or the type of percolation (site, bond or even continuum)^47^. In other words, it is only in the presence of long-range correlations that the critical exponents of percolation transition can be changed^50^. Therefore, the different critical exponents observed in the percolation of force-bearing particles are suggestive of a specific type of long-range correlations, which we speculate is directly related to the emergence of long-range stress correlation in thermal amorphous solids.

We expect that the above analyses of the force-bearing network are relevant for colloidal and granular systems in which the interparticle interactions are dominated by finite-range repulsions. A generalisation to systems interacting with long-range attractions is not immediately straightforward. However, we have performed analyses in systems with the Lennard-Jones potential by focusing on the strong interacting bonds (see Supplementary Figs. 13 and 14 and discussions accordingly). The preliminary results positively support the effectiveness of our approach, therefore pointing to its general relevance in the understanding of amorphous materials. This situation is somewhat reminiscent of the jamming transition, which is not directly inaccessible in systems with Lennard–Jones interactions^51,52^ but still relevant in the understanding of properties such as the boson peak^53^.

### Emergence of non-zero shear modulus

The shear modulus is a central mechanical property of condensed matter, which has been intensively studied as an important characteristic of the liquid-to-glass transition^15–17,54,55^. As has been discussed in ref. ^56^, the apparent shear modulus physically originates from the constraint in the configuration space, meaning that its value actually relies on the time scale used in the calculation of Eqs. (2)–(5) in ‘Methods’. To properly probe the shear modulus representative of the ensembles that are generated using different cooling rates (see ‘Methods’; after each step of the quench, the system is equilibrated for Δ*t*, which controls the cooling rate *γ* = Δ*T*/Δ*t*), we use different sampling times of Δ*t* according to *γ*. Since Δ*t* for *γ* = 10^−3^ is too short for necessary averages, we focus on ensembles with smaller *γ*. At each temperature, 400 trajectories starting from independent configurations are simulated to gain information of the average and the fluctuation of shear modulus.

**Fig. 5:**
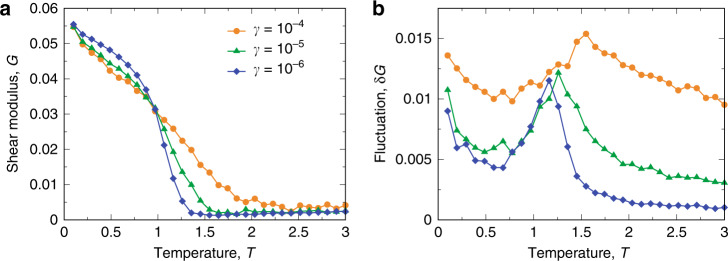
Temperature dependence of shear modulus and its fluctuation. Temperature dependence of the shear modulus *G* (**a**) and its fluctuation *δG* (**b**) for different cooling rates *γ*. The *γ*-dependent nonequilibrium glass transition temperature is signalled by the onset of the growth of *G* and a peak of *δG*. Such a *γ*-dependent (laboratory) glass transition accompanied by the emergence of solidity is consistent with the characterization of spatial stress correlations shown in Fig. 1c.

Figure 5a shows the temperature dependence of the average *G* for several cooling rates. As expected, the onset of the non-zero *G* depends on *γ*, which is consistent with the onset of the nontrivial shear-stress correlation (see Fig. 1c). Therefore, the long-range stress correlation and the non-zero shear modulus are expected to be the closely related mechanical facets of the rigidity of amorphous solids. Interestingly, the fluctuation (standard derivation) of the shear modulus *δG* of the corresponding ensemble shows a peak at the nonequilibrium glass transition temperature, as shown in Fig. 5b. This means that strong fluctuations of the system exist among solid-like and liquid-like configurations, in line with a recent study in polymer glasses^54^. Therefore, this result further supports that the solidity emerges as a result of the nonequilibrium glass transition, which is not related to any underlying thermodynamic phase transition.

## Discussion

In summary, our results demonstrate the emergence of long-range stress correlations in thermal amorphous solids even under giant anharmonic effects, which provides fresh insight for the understanding of disordered solid materials. We show that the long-range stress correlations emerge as a result of the protocol-dependent (laboratory) glass transition when the system falls out of equilibrium, which is hence intrinsically nonequilibrium in nature and not related to any hypothesised thermodynamic phase transition. Fundamentally, the giant anharmonic effects are shown to be related to the fractal-like structure of the configuration space (PEL), which characterises amorphous solids as peculiar states of matter, entirely different from the crystalline counterparts. We propose that the nonequilibrium glass transition is accompanied by mechanical self-organisation towards “partial mechanical equilibrium”, namely the effective mechanical equilibrium realised in a subset of particles. We demonstrate that the percolation of underlying force-bearing particles serves as the physical foundation of the observed long-range stress correlations. Such a percolation transition of force-bearing particles is well-defined in the large system-size limit, providing a novel mechanical rationale of the nonequilibrium glass transition. All these findings may open up a way towards the fundamental understandings of thermal amorphous solids and nonequilibrium glass transition from the mechanical perspective.

It would be an interesting step forward to directly explore the long-range stress correlation and especially the percolation of the force-bearing network in experiments with single-particle resolutions. With the significant advances in super-resolution optical microscopy in recent years, both from experimental techniques^57,58^ and image-processing methods^59^, such measurements can be realised in colloidal systems with high-speed confocal microscopy^60^. The experimental setup which has been widely used in the study of vibrational modes in colloidal systems may be easily adjusted for this purpose^61–64^. Besides, the photoelastic granular system^45^ and the emulsion system^13^ under external vibration, where force chains can be determined, respectively, through photoelastic pattern and shape distortions, may also be used as platforms for these measurements. The generalisation of our analysis from simple glass formers to those with complex interactions, e.g., glasses with directional bonding or long-range interactions^2^, is another interesting direction to explore in future studies. Stability of space-spanning mechanical network in amorphous solids should play a key role in various phenomena such as ageing and devitrification^65^ and mechanical fracture. Recently, a similar scenario has been reported for the emergence of elasticity in colloidal gels due to the mechanical percolation^66,67^. These findings seem to indicate a general origin of apparent rigidity in disordered nonergodic materials made of particles covering the glassy and gel states. They would shed new light on not only the fundamental relationship between nonergodicity and rigidity but also the commonality and difference between glasses and gels.

## Methods

### Models and simulation methods

We employ systems consisting of a 50:50 binary mixture of elastic particles, which have been widely studied as a paradigmatic model for amorphous solids and also serve as canonical glass formers^51,52,68^. We mainly study the harmonic repulsive systems, in which the interaction between particles *i* and *j* is given by $V({r}_{ij})=\epsilon {(1-{r}_{ij}/{\sigma }_{ij})}^{2}/2$, when *r*_*ij*_ < *σ*_*ij*_ and zero otherwise. Here *r*_*ij*_ is the separation between the two particles and *σ*_*ij*_ is the sum of their radii. Large and small particles have a diameter ratio 1.4 but the same mass *m*^52,68^. The length, mass, energy and time are in units of small particle diameter *σ*, *m*, *ϵ* and $\sqrt{m{\sigma }^{2}/\epsilon }$. The temperature is in the unit of 10^−3^*ϵ*/*k*_B_, with the Boltzmann constant *k*_B_ set to unity. We mainly focus on two-dimensional (2D) systems for the sake of computational efficiency, with which we can test carefully the parameter dependence of results, the finite-size effects, the convergence of numerics, and also performed additional analysis of shear modulus. Systems ranging from *N* = 512 to 262,144 particles are used to track finite-size effects, and we mainly present results of *N* = 4096, unless otherwise specified. The volume fraction is fixed at *ϕ* = 0.91 [$=\mathop{\sum }\nolimits_{i = 1}^{N}\pi {\sigma }_{i}^{2}/4{L}^{2}$] so that the systems have well-defined inherent states at zero temperature, and the glass transition is controlled by temperature. Molecular dynamics simulations are performed in square boxes in 2D and cubic boxes in 3D, with periodic boundary conditions in the *NVT* ensemble.

To check the general relevance of our conclusions, we have studied the following systems for selected properties, all of which are 50:50 binary mixtures of spherical particles with the size ratio of 1.4: (1) 2D harmonic systems of different volume fractions *ϕ* = 0.86, 0.95 and 1.0 with the glass transition driven by temperature; (2) 2D harmonic systems at fixed temperature *T* = 1.0 with the glass transition driven by the increase of the volume fraction *ϕ*; (3) 3D harmonic systems with fixed volume fraction *ϕ* = 0.7 [$=\mathop{\sum }\nolimits_{i = 1}^{N}\pi {\sigma }_{i}^{3}/6{L}^{3}$] and controlled by temperature; and (4) 2D systems with Lennard-Jones interactions at number density *ρ* = 0.61 and controlled by temperature. More details are given in the Supplementary Note 2.

Configurations in mechanical equilibrium (*T* = 0), i.e. the inherent structures, are generated by relaxing the finite-temperature systems to the nearest potential energy minima using the fast inertial relaxation engine (FIRE) algorithm^39^.

### Protocols to generate the glass state

To follow the evolution of stress correlations in the temperature range from the simple-liquid regime to deep in the solids, we construct the following scheme to mimic the formation of glasses by cooling in experiments. The systems are first equilibrated at high temperature (*T* = 3.0 with *τ*_*α*_ ≈ 47) for *t* = 10,000 and then cooled down to *T* = 0.1 in a stepwise fashion. The cooling rate is defined as *γ* = Δ*T*/Δ*t*, where the temperature step is fixed to Δ*T* ≈ 0.1 and Δ*t* is the equilibration time at each temperature point, which controls *γ*. Ten configurations with a time interval of *dt* = 5 are sampled at the end of equilibration for each step of cooling. This simulation setup ensures the generation of well-defined ensembles with the degree of annealing controlled by *γ*. Because enhanced statistics is necessary to resolve the power-law correlation at long distances and temperatures close to the glass transition (see Supplementary Fig. 5), as many as 2000 independent cooling trajectories are simulated, covering a range of cooling rates from *γ* = 10^−3^ to 10^−6^. We note that, particularly when entering the glassy regime, even extremely long-time average does not work as efficiently as our method to sample the configuration space. For extremely low temperatures below *T* = 0.1, the systems are directly quenched from *T* = 0.1 to the target temperature and then equilibrated for Δ*t* = 10^4^, and configurations are sampled at the end of equilibration. Basic characterisations of glassy dynamics are given in the Supplementary Figs. 1–3, providing two characteristic temperatures of the system: the ideal glass transition temperature *T*_0_ ≈ 0.63 from the Vogel–Fulcher–Tammann fitting of relaxation time *τ*_*α*_, the onset temperature *T*_on_ ≈ 2.1 from the crossover between the Arrhenius and non-Arrhenius behaviours, and the mode-coupling temperature *T*_mct_ ≈ 1.21^37^. Accordingly, the temperature dependence of shear-stress correlations (see below) shown in Fig. 1c indicates that our simulations have accessed nonequilibrium glass transition with *T*_g_ lying both above and below the mode-coupling temperature. This is comparable to the dynamic range covered by present-day colloidal experiments^69,70^.

Another protocol quite often used to generate a glass is to quench the system directly from the high-temperature liquid state to the target low temperature. We have also studied how the stress correlation evolves in this situation, which is particularly relevant for the understanding of the long-range character as an emergent property of the metastable glass states. See Supplementary Fig. 7 and discussions accordingly for details.

### Definition of particle-level shear stress

The particle-level shear stress for particle *i* can be defined as ${\sigma }_{xy}^{i}=\frac{1}{{V}_{i}}{\sum }_{j}{f}_{ij}^{x}{r}_{ij}^{y}/2$, where *V*_*i*_ is the local volume (area in 2D) of particle *i*, **f**_*ij*_ is the interacting force between particles *i* and *j*, *x* and *y* indicate the corresponding Cartesian components, and the summation goes over all contacting neighbours of particle *i*^8,71^. *V*_*i*_ can be calculated according to the Voronoi tessellation^72^, but in practice, we set *V*_*i*_ to unity which does not alter the results^8,71^. The macroscopic shear stress *Σ*_*xy*_ can then be written as the average of the contributions from individual particles ${\Sigma }_{xy}=\mathop{\sum }\nolimits_{i = 1}^{N}{V}_{i}{\sigma }_{xy}^{i}/V$, where *V* represents the total area of the system in 2D and volume in 3D. Here the kinetic term is neglected since it trivially contributes only to short-range fluctuations when considering the spatial stress correlations^8^. Alternative definitions can be found in the literature^3,8,13^, which however do not affect the long-range stress correlations of interest.

### Characterisation of force-bearing networks

To access the formation of effectively stable force networks in thermal amorphous solids, we identify force-bearing particles constrained by strong force contacts. An important observation is that particles essential in the backbone of the force network are necessarily connected by at least two strong force bonds^46^. Therefore, we quantify the degree of local force balance for each particle using the remaining force rescaled by the strength of its two strongest force bonds: ${F}_{{\rm{str}}}^{i}=| {\sum }_{j}{{\bf{f}}}_{ij}| /\langle {f}_{i}\rangle$, where **f**_*ij*_ is the force acting on particle *i* from *j*, and 〈*f*_*i*_〉 is the averaged strength of the two strongest force bonds of particle *i*. Particles with ${F}_{{\rm{str}}}\,<\,1$ are expected to contribute to the stability of force networks. To diminish the influence of thermal noises, we monitor the system over a fast *β* (or, cage-rattling) time scale (*t* = 50) and particles with ${F}_{{\rm{str}}}\,<\,1$ for a probability higher than 50% are categorised as force-bearing ones. In a related vein, similar ideas for the construction of effective force network in partial mechanical equilibrium were successfully implemented in the study of jamming transition at finite temperatures^73–75^.

Upon cooling, the fraction of force-bearing particles increases and gradually a system-spanning network is formed below some critical temperature. We carry out standard percolation analysis of the clustering of force-bearing particles in this process^47^. The occupation fraction *f* is defined as the fraction of the identified force-bearing particles. Percolation transition is identified whenever a cluster spans from one side of the system to the other in any direction. The percolation probability at *T* or the corresponding occupation fraction *f* is defined as the proportion of configurations in the ensemble which contain percolating clusters. We perform the percolation analysis for ensembles generated by different cooling rates. For *γ* = 10^−3^ and 10^−4^, different system sizes from *N* = 512 to 16,384 are used for the finite-size scaling analysis; whereas for *γ* = 10^−5^ and 10^−6^, bounded by the computational power, systems with *N* = 512 to 4096 particles are analysed.

### Characterisation of shear modulus

We characterize the behaviour of shear modulus upon cooling across the non-equilibrium (laboratory) glass transition, in parallel with the study of spatial shear-stress correlations. The elastic constants at finite temperatures are calculated from the fluctuation–dissipation theorem as follows^56,76^:

1 $${C}_{\alpha \beta \gamma \kappa }={C}_{\alpha \beta \gamma \kappa }^{{\rm{B}}}-{C}_{\alpha \beta \gamma \kappa }^{{\rm{S}}}+{C}_{\alpha \beta \gamma \kappa }^{{\rm{K}}},$$

where

2 $${C}_{\alpha \beta \gamma \kappa }^{{\rm{B}}}=\frac{1}{V}\sum _{i<\,j}\left\langle \left(\frac{{\partial }^{2}U}{\partial {{r}^{ij}}^{2}}-\frac{1}{{r}^{ij}}\frac{\partial U}{\partial {r}^{ij}}\right)\frac{{r}_{\alpha }^{ij}{r}_{\beta }^{ij}{r}_{\gamma }^{ij}{r}_{\kappa }^{ij}}{{{r}^{ij}}^{2}}\right\rangle ,$$

3 $${C}_{\alpha \beta \gamma \kappa }^{{\rm{S}}}=\frac{V}{{k}_{{\rm{B}}}T}\left[\left\langle \sum_{\alpha \beta }\sum_{\gamma \kappa }\right\rangle -\left\langle \sum_{\alpha \beta }\right\rangle \left\langle \sum_{\gamma \kappa }\right\rangle \right],$$

4 $${C}_{\alpha \beta \gamma \kappa }^{{\rm{K}}}=2\rho {k}_{{\rm{B}}}T({\delta }_{\alpha \gamma }{\delta }_{\beta \kappa }+{\delta }_{\alpha \kappa }{\delta }_{\beta \gamma }),$$

5 $${\Sigma }_{\alpha \beta }=\rho {k}_{{\rm{B}}}T{\delta }_{\alpha \beta }-\frac{1}{V}\sum _{i<\,j}\left(\frac{\partial U}{\partial {r}^{ij}}\right)\frac{{r}_{\alpha }^{ij}{r}_{\beta }^{ij}}{{r}^{ij}}.$$

Here the subscripts *α*, *β*, *γ* and *κ* indicate the Cartesian components, the superscripts *i*, *j* are particle indices, *V* is the area in 2D and the volume of the system in 3D, *ρ* is the number density, *U* is the total potential energy function, *r*^*ij*^ is the separation between two interacting particles *i* and *j*, and 〈 ⋅ 〉 indicates the ensemble average. In particular, ${C}_{\alpha \beta \gamma \kappa }^{{\rm{B}}}$ is the so-called Born term from affine deformations, ${C}_{\alpha \beta \gamma \kappa }^{{\rm{S}}}$ is the nonaffine component, and ${C}_{\alpha \beta \gamma \kappa }^{{\rm{K}}}$ is contributed by the kinetic energy, which is found to be much smaller than the other terms in amorphous solids^76^. In this work, we focus on the shear modulus *G* = *C*_*xyxy*_.

## Supplementary information

Supplementary Information

## Data Availability

The data that support the findings of this study are available from the corresponding author upon reasonable request.

## Code availability

The codes that are used to generate results in the paper are available from the corresponding author upon reasonable request.
