# Supplementary material for: Emergent solidity of amorphous materials as a consequence of mechanical self-organisation
Source: Nat Commun. 2020 Sep 25;11:4863. doi: 10.1038/s41467-020-18663-7 (PMC7519136; doi:10.1038/s41467-020-18663-7)
Supplement: Supplementary file 1 — Supplementary Information [file 41467_2020_18663_MOESM1_ESM.pdf]

Supplementary Information for "Emergent solidity of amorphous materials as a consequence of mechanical self-organisation"

Hua Tong, Shiladitya Sengupta, and Hajime Tanaka

SUPPLEMENTARY NOTE 1. ADDITIONAL RESULTS FOR 2D HARMONIC SYSTEMS WITH $\phi = 0.91$

1. Basic analysis of glassy dynamics. Here we characterize the structure relaxation in glass-forming liquids [1]. In particular, we show results of the 2D harmonic systems with $N = 4096$ particles focused in this work. We use the position of particle j relative to its n_j neighboring particles l, $\underline{\mathbf{r}}_j(t) = \mathbf{r}_j(t) - \sum_l \mathbf{r}_l(t)/n_j$, to characterize the dynamics in 2D [2, 3], which helps to remove the long-wavelength Mermin-Wagner fluctuations [3–6]. Then the self part of the intermediate scattering function is given as

$$F_s(k,t) = \left\langle \frac{1}{N} \sum_j \exp\left(i\mathbf{k} \cdot [\underline{\mathbf{r}}_j(t) - \underline{\mathbf{r}}_j(0)]\right) \right\rangle . \tag{1}$$

Here $\underline{\mathbf{r}}_j(t)$ is the relative position of particle j at time t, $\mathbf{k}$ is the scattering vector with $k = |\mathbf{k}|$ close to the first peak of the static structure factor, and brackets indicate an ensemble average. The mean square displacement (MSD) is another probe of the dynamics:

$$\langle \Delta r^2(t) \rangle = \left\langle \frac{1}{N} \sum_j [\underline{\mathbf{r}}_j(t) - \underline{\mathbf{r}}_j(0)]^2 \right\rangle . \tag{2}$$

Figure 1 shows $F_s(k,t)$ and MSD for a wide range of temperature. The emergence of the intermediate-time plateau of both characterisations in the supercooled regime signals the transient localisation, i.e., caging, a universal dynamic feature of supercooled liquids.

The structure relaxation time τ_α is measured from the time decay of the self-intermediate scattering function: $F_s(k, \tau_\alpha) = e^{-1}$. Figure 2 shows the temperature dependence of τ_α. From Fig. 2a, we find that the drastic dynamical slowing down is well described by the Vogel-Fulcher-Tammann (VFT) relation $\tau_\alpha = \tau_0 \exp[DT_0/(T - T_0)]$, which suggests a hypothetical divergence of τ_α at the ideal glass transition temperature $T_0 \approx 0.63$. We may also plot τ_α as a function of the inverse of temperature $1/T$, as shown in Fig. 2b, from which a crossover from the Arrhenius to non-Arrhenius behaviours is identified as the onset temperature $T_{\mathrm{on}} \approx 2.1$ of sluggish glassy dynamics. The appearance of

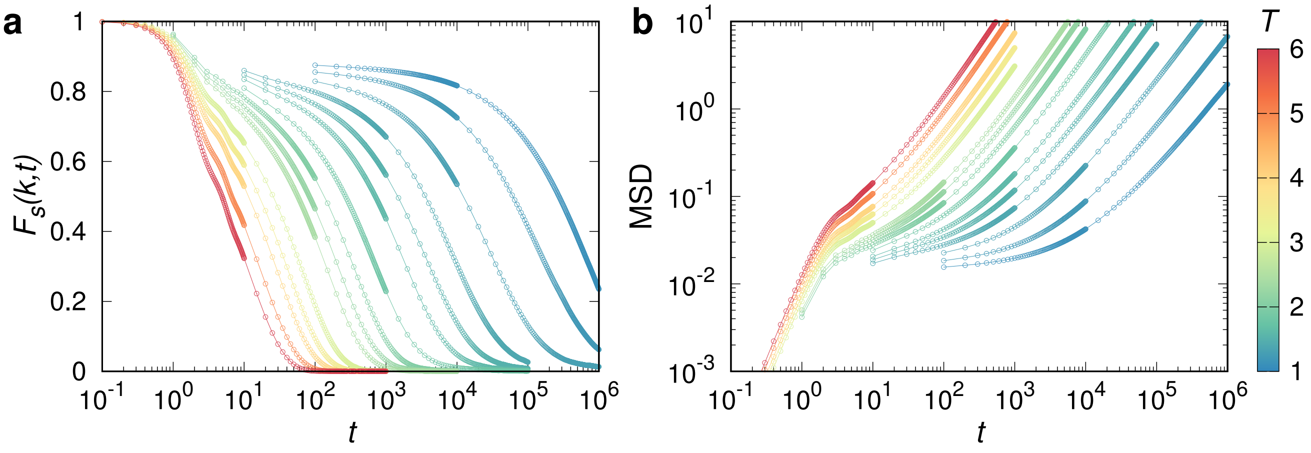

Supplementary Figure 1: **Self-intermediate scattering function and mean square displacement. a,** Temperature dependence of Self-intermediate scattering function $F_s(k,t)$. **b,** Temperature dependence of MSD. The data colour changes from red to blue with a decrease in T.

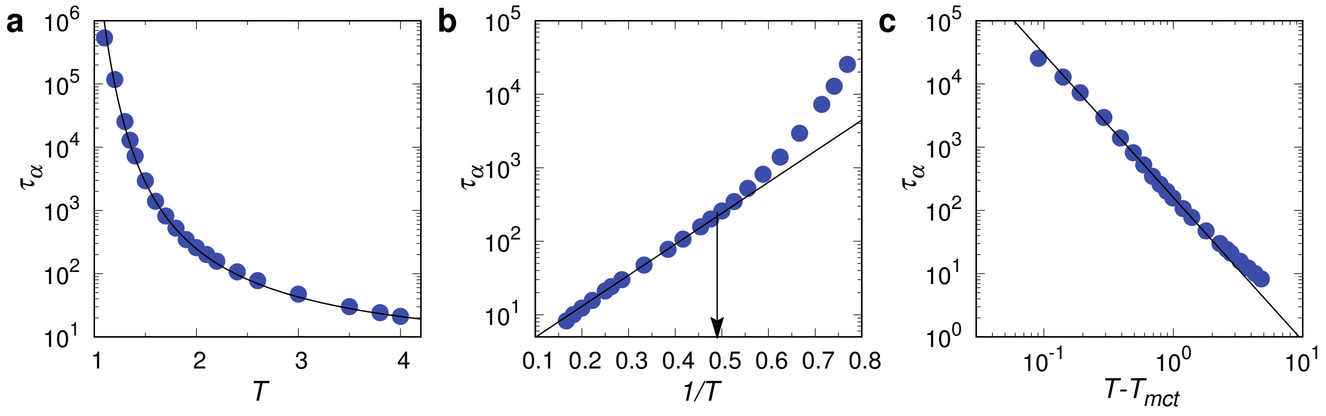

Supplementary Figure 2: **Temperature dependence of the structure relaxation time** τ_α**. a,** VFT fitting of the temperature dependence of τ_α. The solid line is a fitting according to the VFT law $\tau_\alpha = \tau_0 \exp[DT_0/(T - T_0)]$, from which we extract the ideal glass transition temperature $T_0 \approx 0.63$. **b,** Arrhenius fitting to the high-temperature data of τ_α. The solid line is a fitting to the Arrhenius behavior $\tau_\alpha \sim \exp(\Delta E/T)$. The crossover where τ_α starts to deviate from the Arrhenius behavior is identified as the onset temperature of sluggish glassy dynamics, $T_{\rm on} \approx 2.1$, as indicated by the arrow. **c,** Power-law fitting of the temperature dependence of τ_α. The solid line shows a fitting according to the mode-coupling theory (MCT) $\tau_\alpha \sim (T - T_{\rm mct})^{-\delta}$, with $T_{\rm mct} = 1.21$ being the estimated MCT temperature.

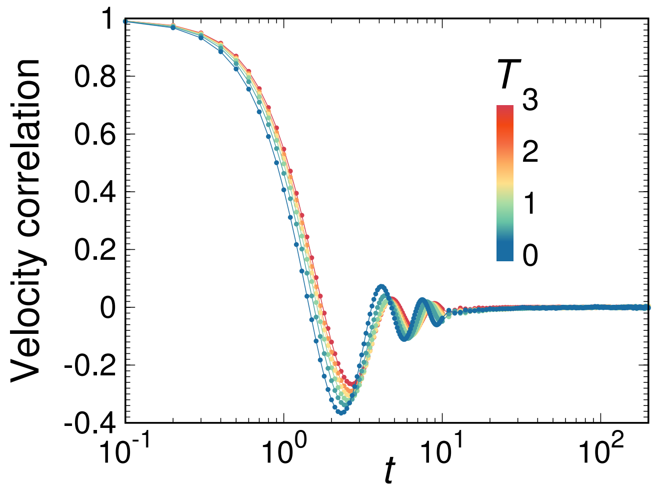

Supplementary Figure 3: **Temperature dependence of velocity correlation** $\langle \mathbf{v}(t) \cdot \mathbf{v}(0) \rangle$**.** Here $\mathbf{v}(t) = \{\mathbf{v}_i(t)\}$ is the velocity field of the whole system. From the relaxation curves, the period of oscillation $\tau_o \approx 5$ can be identified, which has a weak temperature dependence.

the non-Arrhenius behaviour suggests a growth of the activation energy, which is an indication of cooperative dynamics typical for the so-called "fragile" glass-forming liquids. Figure 2c further shows a power-law fitting of the temperature dependence of τ_α, as predicted by the mode-coupling theory (MCT). The MCT temperature is empirically estimated to be $T_{\rm mct} = 1.21$.

In addition, we characterise the velocity correlation in a temperature range covering both simple liquids and deep in the solid phase, as shown in Fig. 3. From the oscillation of velocity correlation, we estimate the period of oscillation of the short-time vibrational motions $\tau_o \approx 5$, which shows only a weak temperature dependence.

2. Examination of finite-size effects. Here we examine the finite-size effect on the long-range stress correlation in thermal amorphous solids. In principle, the long-range character cannot be *proven* but rather *suggested* by numerical simulations with finite-size systems. In this context, the method of finite-size scaling, used extensively in numerical studies of critical phenomena, is particularly suited for investigations of the emergence of the long-range correlations.

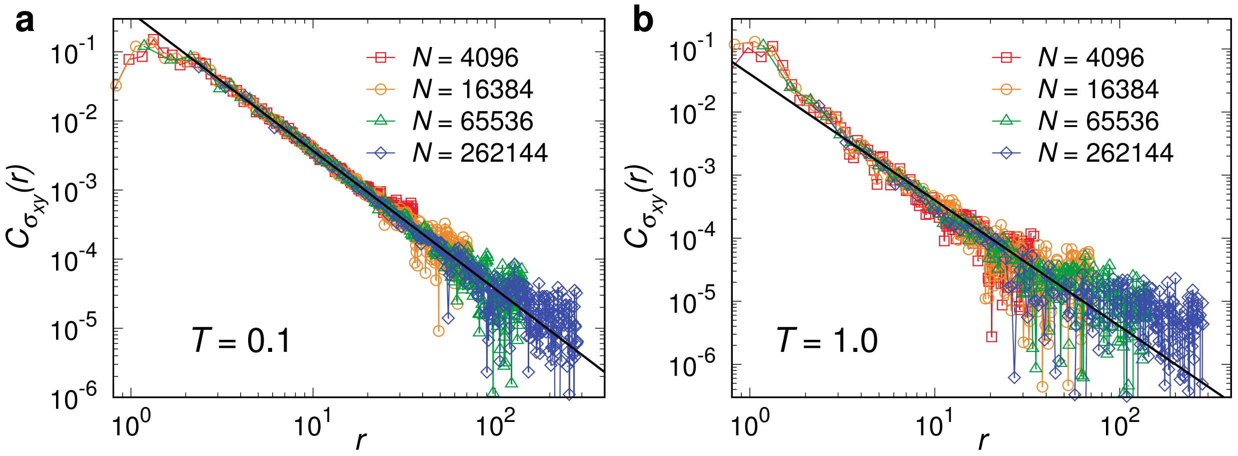

Supplementary Figure 4: **Finite-size analysis of the shear-stress correlation. a,** Angle-averaged shear-stress correlation $C_{\sigma_{xy}}(r)$ at a low temperature ($T = 0.1$) for different system sizes ranging from $N = 4096$ to $N = 262144$. **b,** The same analysis as **a**, for a higher temperature ($T = 1.0$), slightly below the experimental glass transition temperature. Here the ensemble of configurations are generated by a constant cooling rate $\gamma = 10^{-4}$ but the results are insensitive to the choices of γ. Consistent results are confirmed for different system sizes, following the power-law function $1/r^2$ as indicated by the solid lines.

In the main text (Fig. 4), we show through finite-size scaling that a well-defined percolation transition of the force-bearing particles exists in the large system size limit. The result also suggests that the disordered solid states below the non-equilibrium glass transition temperature are not affected by system sizes, so that a general relation between the long-range stress correlation and the percolating force-bearing network is observed. Here we further illustrate this point in Fig. 4. The angle-averaged shear-stress correlation $C_{\sigma_{xy}}(r)$ for a range of system sizes from $N = 4096$ to $N = 262144$ are plotted, which in turn correspond to linear sizes from $L \approx 72$ to $L \approx 579$. As we can see, $C_{\sigma_{xy}}(r)$ from a smaller system closely matches those from larger ones. This suggests that finite-size effects can be neglected in the characterisation of stress correlations in the solid phase. Therefore, we mainly focus on the systems with $N = 4096$ particles, unless otherwise specified, in order to extensively explore a wide range of cooling rates, the convergence of numerics with very large ensembles, and additional properties like the shear modulus.

3. The necessity of large ensemble. It is worth pointing out that the decay of power-law correlation functions $1/r^d$ in d dimensions is relatively fast from the viewpoint of numerics. In order to obtain clear evidence of such long-range correlations, a large ensemble of *independent* configurations are necessary to suppress statistical fluctuations. We note that, particularly when entering the glassy regime, even extremely long-time average does not work so efficiently to sample the configuration space. To emphasise this point, we compare the results calculated by using a different number of configurations in Fig. 5. At low temperature ($T = 0.1$) deep in the solid phase, we find that the data from a smaller ensemble of configurations is more fluctuated, although the power-law behaviour is still noticeable (Fig. 5a). At higher temperature ($T = 1.0$), slightly below the γ-dependent T_g, we see that while the power-law behaviour is still clear for a large ensemble of configurations, it is basically hidden behind the noise for a small ensemble of configurations (Fig. 5b). In Fig. 5c, we further show $C_{\sigma_{xy}}(r)$ from an ensemble of $N_{\rm conf} = 100$ configurations for a range of temperatures covering both above and below $T_g \approx 1.3$ (see Fig. 1c in the main text). The results are essentially indistinguishable for different temperatures, so the crossover at T_g cannot be resolved. However, if the power-law scaling is plotted unadvisedly together with the data as in Fig. 5c, the long-range character may be incorrectly claimed (we note that this can be found in the literature).

4. Additional information on the giant anharmonic effects from pressure. In the main text, we have characterised the giant anharmonic effects from the perspective of PEL by focusing on the distance and potential energy with respect to IS during energy minimisations (see Figs. 2b and 2c in the main text). Here we further characterise the potential pressure (pressure without the kinetic component) to gain more insights from the aspect of internal stress. To be specific, we focus on the difference of potential pressure between the configuration on the way of energy minimisation and the IS: $\Delta P = |P - P^{\rm IS}|$, and results are shown in Fig. 6. As expected, a linear relation

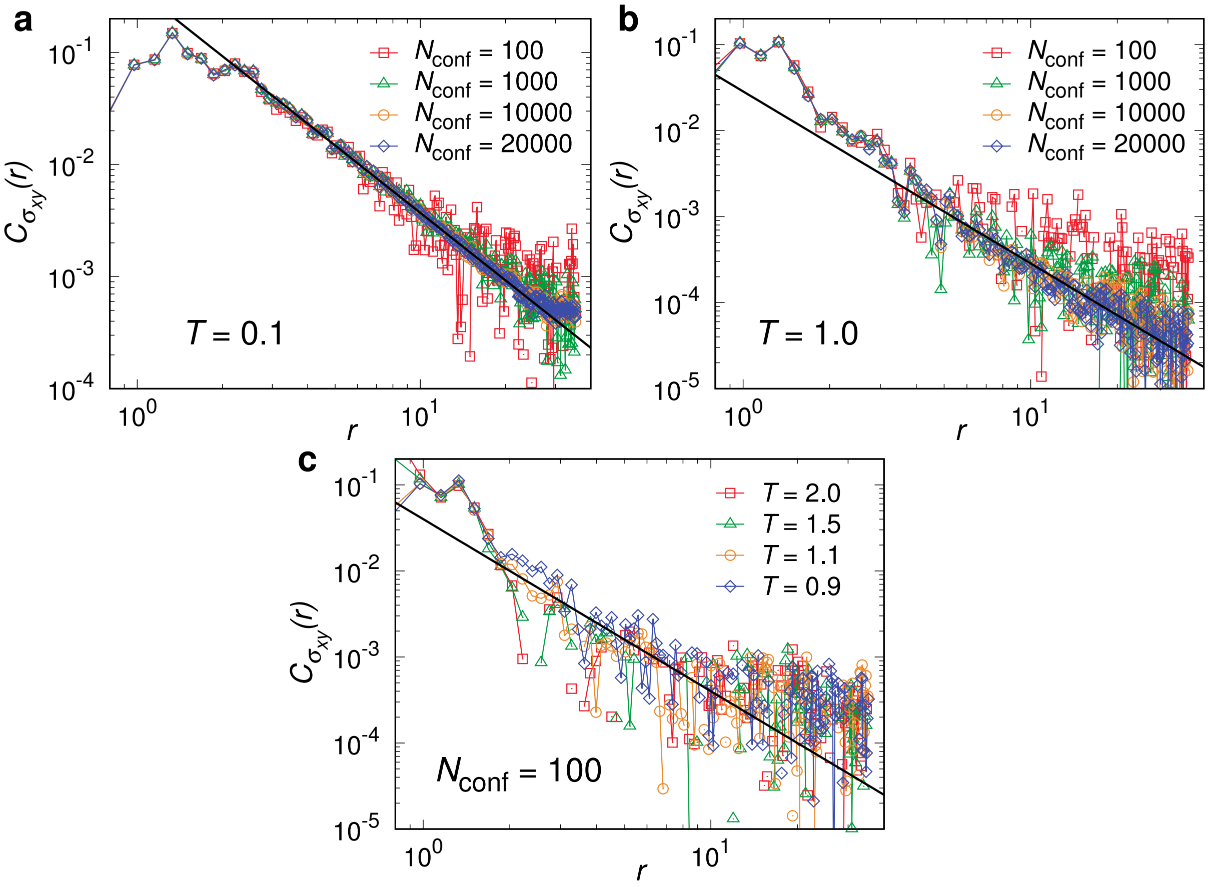

Supplementary Figure 5: **Examination of ensemble-size dependence of the shear-stress correlation. a,** Angle-averaged shear stress correlation $C_{\sigma_{xy}}(r)$ at a low temperature ($T = 0.1$), obtained by averaging over different numbers of configurations $N_{\rm conf}$. **b,** The same analysis as **a** for a higher temperature ($T = 1.0$), slightly below the experimental glass transition temperature. **c,** $C_{\sigma_{xy}}(r)$ at different temperatures, obtained by averaging over $N_{\rm conf} = 100$ configurations. Here the ensemble of configurations are generated by a constant cooling rate $\gamma = 10^{-5}$ but the results are insensitive to the choices of γ. The solid lines in the panels indicate the power-law scaling $1/r^2$.

$\Delta P \sim \Delta r$ is observed for small Δr, which is suggestive of a harmonic regime close to IS. A strong deviation can be observed when the system is slightly away from IS, exhibiting similar behaviours as the potential energy shown in Fig. 2c in the main text. Therefore, this observation further confirms the giant anharmonic effects in thermal amorphous solids.

5. Initial development of long-range stress correlation after an instant quench. Until now, we have focused on the static properties related to the stress correlations in amorphous solids. For a better understanding of the long-range character as an emergent property of the metastable glass states, it is also of interest to study how it emerges, starting from an intrinsically random state. To this aim, we bring the system from a high-temperature liquid state to the target temperature by an instant quench and follow the evolution of stress correlation during further equilibration. The evolution of $C_{\sigma_{xy}}(r)$ deep in the solid phase after a quench is shown in Fig. 7a. We find that a power-law feature emerges already at $t = 10$ which is in the fast β (or, cage-rattling) time scale and corresponds to only several periods of oscillations (see Figs. 1 and 3). Nevertheless, the correlation continues to grow with time, reflecting the underlying optimisation of the force network for partial mechanical equilibrium. To better quantify this behaviour of time evolution, we follow the integrated shear-stress correlations for a range of target temperatures, as shown in Fig. 7b. In particular, the curve corresponds to Fig. 7a is highlighted with open green circles (second top), which reaches a plateau at around $t = 100$. The same feature is observed for a range of temperatures deep in the solid phase, with the effect of ageing not visible in the current time scale of simulations. The most striking observation here

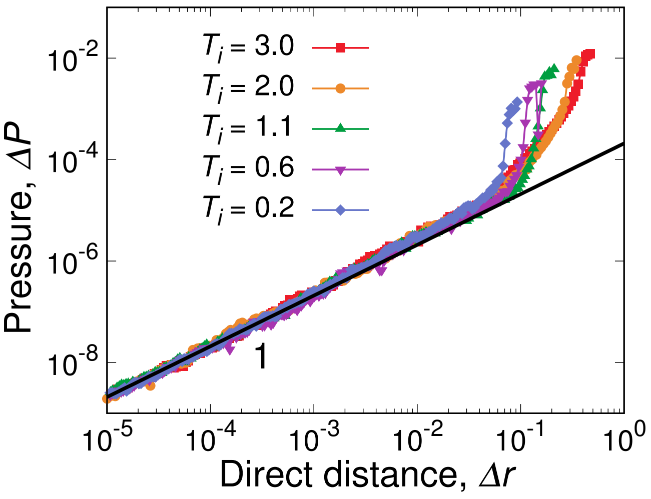

Supplementary Figure 6: **Giant anharmonic effects in thermal amorphous solids seen from pressure.** Corresponding to Figs. 2b and 2c in the main text, here we shown the relationship between the potential pressure difference ΔP and direct distance Δr with respect to IS during energy minimizations for different initial temperatures T_i. The solid black lines indicate the linear relation $\Delta P \sim \Delta r$. Note that the average pressure in IS decreases from 0.023 to 0.021 when the initial temperature is lowered from 3.0 to 0.2.

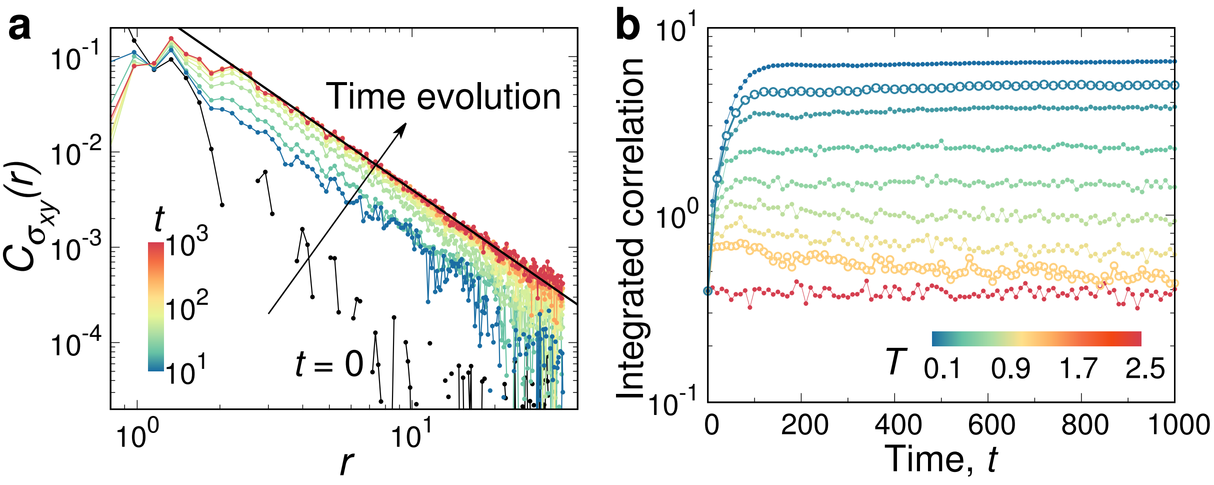

Supplementary Figure 7: **Initial development of long-range stress correlation. a,** Evolution of angle-averaged shear stress correlation $C_{\sigma_{xy}}(r)$. The configurations are generated by instant quench of equilibrated ones at $T = 3$ (liquid regime) to the target temperature $T = 0.1$ (solid regime) and then evolved with time in the NVT ensemble. The initial correlation at $t = 0$ is in black colour. Approximate power-law correlation emerges at very short time $t \approx 10$ and optimizes with time. The black solid line is a fit to power law $1/r^2$ for data at $t = 1000$. **b,** Time evolution of integrated shear-stress correlations at different target temperatures, covering liquid to deeply solid regimes. The evolution corresponds to panel **a** is shown with open circles in green colour (second top). The evolution at $T = 1.2$ is highlighted with open circles in orange colour (second bottom), which shows a gradual decay at long times, the signature of aging.

is the surprisingly short time scale required for the development of long-range (or quasi-long-range) stress correlations, which strongly suggests their relevance when considering the properties of realistic metastable glassy states [7]. This result indicates that the establishment of partial mechanical equilibrium after an instant quench in temperature, i.e., solidification, is a fast process facilitated by vibrational motions rather than diffusive motions. It also validates that our simulations, which cover much longer time scales, are adequate to study this process.

At temperatures close to the non-equilibrium glass transition, we find nonmonotonic evolutions of the correlation, which is a clear sign of the ageing effect. This is most clearly seen from the curve of open orange circles at $T = 1.2$

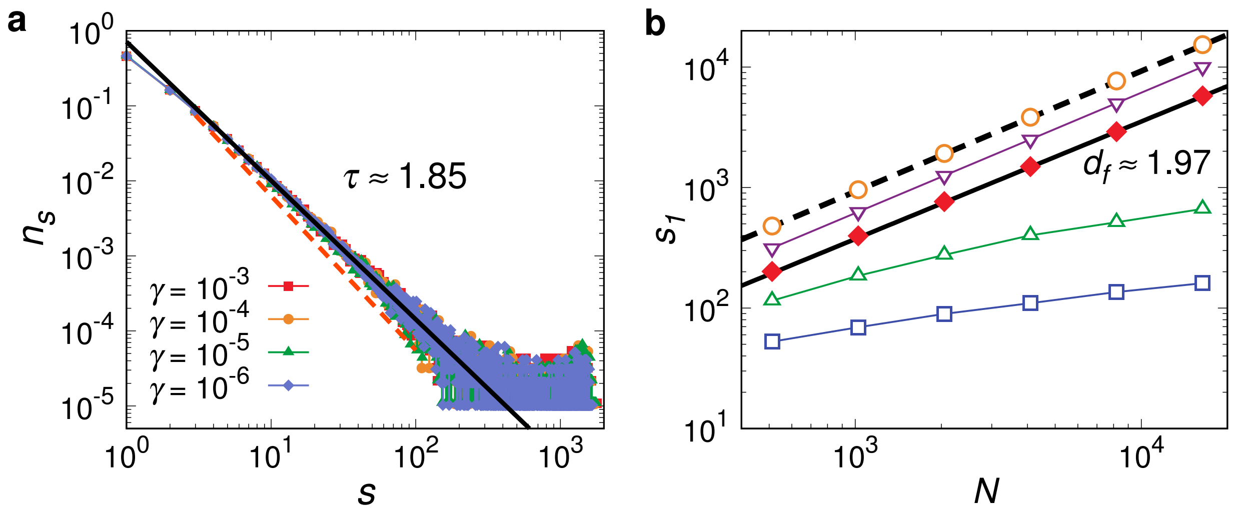

Supplementary Figure 8: **Analysis of the force-bearing network at the percolation transition. a,** Probability distribution n_s of cluster sizes s at the critical temperature or occupation fraction f_c for different γ. The solid line shows a power-law fit according to $n_s \sim s^{-\tau}$ with $\tau = 1.85$. The agreement of the data with the random-percolation exponent $\tau_{\rm rand} = 187/91$, indicated by the dashed line, is significantly worse. **b,** Size of the largest cluster s_1 versus the system size N for $\gamma = 10^{-4}$ across the percolation transition. Temperature increases from top to bottom ($T = 0.1$, 0.6, 1.1, 1.4, and 2.5), with the data at the critical temperature $T_c = 1.1$ indicated by filled diamonds and the others by open symbols. The solid line is a fit to $s_1 \sim N^{d_f/d}$, with the fractal dimension of the percolating cluster $d_f \approx 1.97$. The dashed line shows the relation $s_1 \sim N$, reflecting the effectively compact nature of the percolating force-bearing network inside the solid phase.

in Fig. 7b (see Fig. 1c in the main text that at this temperature, the system is expected to go back to the liquid state after long-time relaxations). This result unveils the real-space mechanical aspect of physical ageing, which was previously characterised by geometrical structural changes [8] or macroscopic quantities [9]. This is an interesting point which definitely deserves careful studies in the near future.

6. Further percolation analysis of the force-bearing network. Here we perform further percolation analysis of the force-bearing network, concerning the nature of observed percolation transition within the percolation theory [10]. First, we characterise the probability distribution of cluster sizes at the critical occupation fraction f_c, i.e. at the non-equilibrium glass transition. As shown in Fig. 8, the cluster size shows a power-law distribution $n_s(s) \sim s^{-\tau}$ with $\tau = 1.85$, which is significantly different from the random-percolation exponent $\tau_{\rm rand} = 187/91 \approx 2.05$ [10]. As indicated by the dashed line, such a difference may not be accounted for by numerical uncertainties. It is known that the critical exponents of random percolation are very robust in changing the percolation model details [10, 11]. They do not depend on the short-range structure of the lattice (e.g., square or triangular) or the type of percolation (site, bond or even continuum) [10]. In other words, only in the presence of long-range correlations that the critical exponents of percolation transition can be changed [11]. Therefore, the value of the scaling exponent τ different from $\tau_{\rm rand}$ suggests that the observed percolation of force-bearing particles does not belong to the universality class of random percolation but is controlled by certain long-range correlations. Here, we expect that the emergence of long-range stress correlation in thermal amorphous solids across the non-equilibrium glass transition may be the underlying mechanism of this unusual nature of percolation transition.

We further characterise the topology of the largest cluster across the percolation transition by plotting its size s_1 as a function of the system size N in Fig. 8b. At f_c, as indicated by the filled diamonds and solid line, we find that $s_1 \sim N^{d_f/d}$ with $d_f \approx 1.97$. Within numerical precision, the observed d_f is not different from $d = 2$ (although, within numerical precision, we cannot rule out the possibility that it coincides with $d_{f,\rm rand} = 91/48 \approx 1.9$ of random percolation). This is consistent with the behaviour of the relative size of the largest cluster $\psi = s_1/N$ shown in Fig. 4c of the main text. There, the finite-size scaling suggests an abrupt formation of system-spanning force-bearing network at f_c in the large system size limit with $\psi_c \approx 0.4$. The finite value of ψ_c suggests that the percolating cluster is compact, meaning $d_f \approx 2$. Below f_c, i.e., at high temperatures in the liquid phase, the growth of s_1 does not follow the system size. Therefore, stress correlation should be short-ranged. Above f_c, i.e., at low temperatures in the solid phase, s_1 is proportional to the system size, as expected from a compact nature after (and away from) the percolation

transition.

Finally, we mention that similar observations, namely $\tau < 2$ and $d_f = 2$, have been made in several percolation models, e.g., the no-enclaves percolation [12, 13] and the percolation of cluster holes [14]. In addition, the abrupt formation of a percolating cluster at f_c with a finite $\psi = s_1/N$ is also lively discussed in the so-called "explosive percolation" models [15–19]. Whether there is a deep link between our physical systems and these mathematical models is an interesting topic to be explored in future studies.

1. The emergence of long-range stress correlation in systems with different volume fractions. The emergence of long-range stress correlation driven by temperature is characterised in three more 2D harmonic systems with $\phi = 0.86$, 0.95 and 1.0, in addition to the one with $\phi = 0.91$ on which we have focused in the main text. Figure 9 shows the temperature dependence of integrated shear-stress correlations for all these four systems at a cooling rate of $\gamma = 10^{-5}$. As expected, the non-equilibrium glass transition signalled by the sudden growth of shear stress correlation upon cooling is generally observed, with a higher T_g for larger ϕ. This result confirms that our observation is general with respect to the volume fraction and not restricted to systems close to the jamming transition.

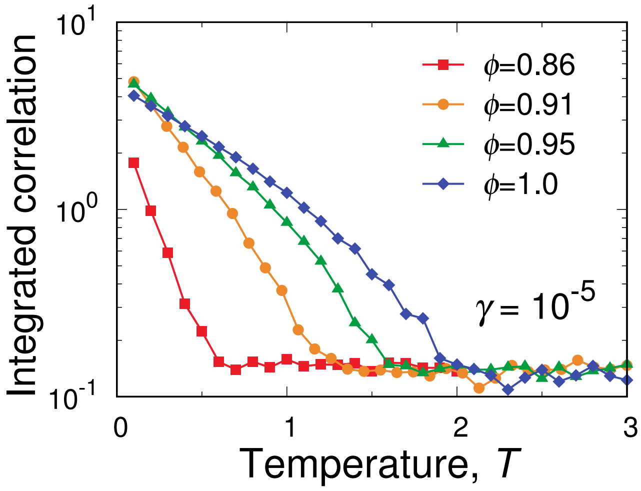

Supplementary Figure 9: **Emergence of long-range stress correlation driven by temperature for different volume fractions.** Temperature dependence of integrated shear-stress correlations for different volume fractions ϕ at a cooling rate $\gamma = 10^{-5}$. The same phenomenology is found that the non-equilibrium glass transition is signaled by the onset of the growth of shear stress correlation at a ϕ-dependent T_g.

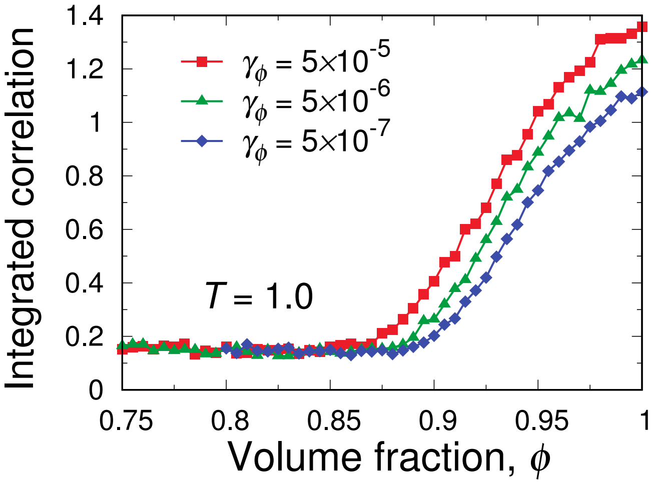

Supplementary Figure 10: **Emergence of long-range stress correlation driven by increasing the volume fraction ϕ, or compression of the total volume V.** Volume fraction dependence of integrated shear-stress correlations for different compression rates γ_ϕ. Temperature is fixed at $T = 1$ during the compression. In comparison with Fig. 1c in the main text, the non-equilibrium glass transition induced by compression shows essentially the same features as that induced by cooling.

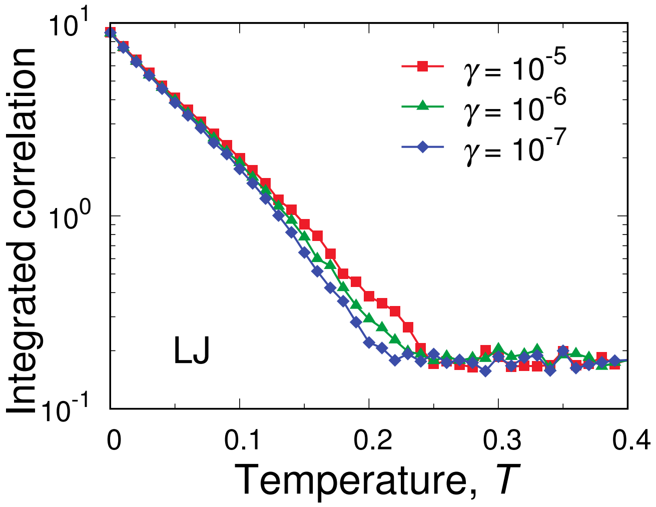

Supplementary Figure 11: **Emergence of long-range stress correlation in 2D Lennard-Jones systems.** Temperature dependence of integrated shear-stress correlations for different cooling rates γ. The γ-dependent non-equilibrium glass transition is signaled by the onset of the growth of shear stress correlation. In comparison with Fig. 1c in the main text for 2D harmonic systems, essentially the same features are observed.

2. The emergence of long-range stress correlation driven by volume fraction. In addition to the non-equilibrium glass transition driven by temperature, which has a close correspondence to that in atomic systems, here we further study the glass transition driven by volume fraction (equivalently, density or pressure), which is more closely related to the colloidal systems [20, 21]. Similar to the case of temperature control (see Methods in the main text), the systems are first equilibrated at $\phi = 0.7$ and $T = 1.0$ for $t = 10000$ and then compressed to $\phi = 1.0$ in a quasi-continuous fashion. The compression rate is defined as $\gamma_\phi = \Delta\phi/\Delta t$, where the compression step is fixed to $\Delta\phi = 5 \times 10^{-3}$ and Δt is the residence time at each volume fraction, which controls γ_ϕ. Figure 10 shows the ϕ-dependence of integrated shear-stress correlations for different compression rates γ_ϕ. It is seen that the nontrivial shear-stress correlation emerges across a γ_ϕ-dependent glass transition, which is essentially similar to that driven by temperature (see Fig. 1c in the main text). This result confirms that the emergence of long-range stress correlation is a general consequence of the non-equilibrium glass transition, no matter it is driven by temperature or volume fraction.

3. Emergence of long-range stress correlation in 2D Lennard-Jones systems. In the main text, we have focused on the system with a finite-range repulsive interaction (see Methods of the main text), which has been widely studied in numerical simulations and shown to behave as canonical glass formers [22, 23]. The simplicity of this model allows us to carry out extensive analyses to explore carefully the parameter dependence of results, the convergence of numerics and the finite-size effects. However, our major findings, that the long-range stress correlation emerges as a consequence of the non-equilibrium glass transition even under giant anharmonic effects, are not restricted to this simplified model but rather generic for glass formers with different interactions. To support this point, we consider an equimolar binary mixture system in which particles i and j interact through a Lennard-Jones (LJ) potential:

$$V(r_{ij}) = 4\epsilon \left[\left(\frac{\sigma_{ij}}{r_{ij}} \right)^{12} - \left(\frac{\sigma_{ij}}{r_{ij}} \right)^{6} \right] + f(r_{ij}), \tag{3}$$

when $r_{ij}/\sigma_{ij} < R_c$ and zero otherwise, where r_{ij} is the particle separation, σ_{ij} is the sum of the particle radii, and $f(r_{ij})$ guarantees that the potential and its first derivative are zero at $r_{ij} = R_c\sigma_{ij}$. Here we set $R_c = 2.5$, particle diameter ratio=1.4, and consider a 2D system with a number density $\rho = 0.61$, which ensures positive pressure at zero temperature. Temperature is in unit of ϵ/k_B. This and similar models have been widely studied as models of atomic glass formers [24–28], e.g., the famous Kob-Anderson model to describe amorphous $Ni_{80}P_{20}$ [24] and the equimolar binary version for metallic glass $Cu_{50}Zr_{50}$ [25, 27, 28].

Figure 11 shows the temperature dependence of integrated shear-stress correlations in LJ systems. Nontrivial stress correlations are found to emerge across the cooling-rate dependent glass transition, with the same features as in the

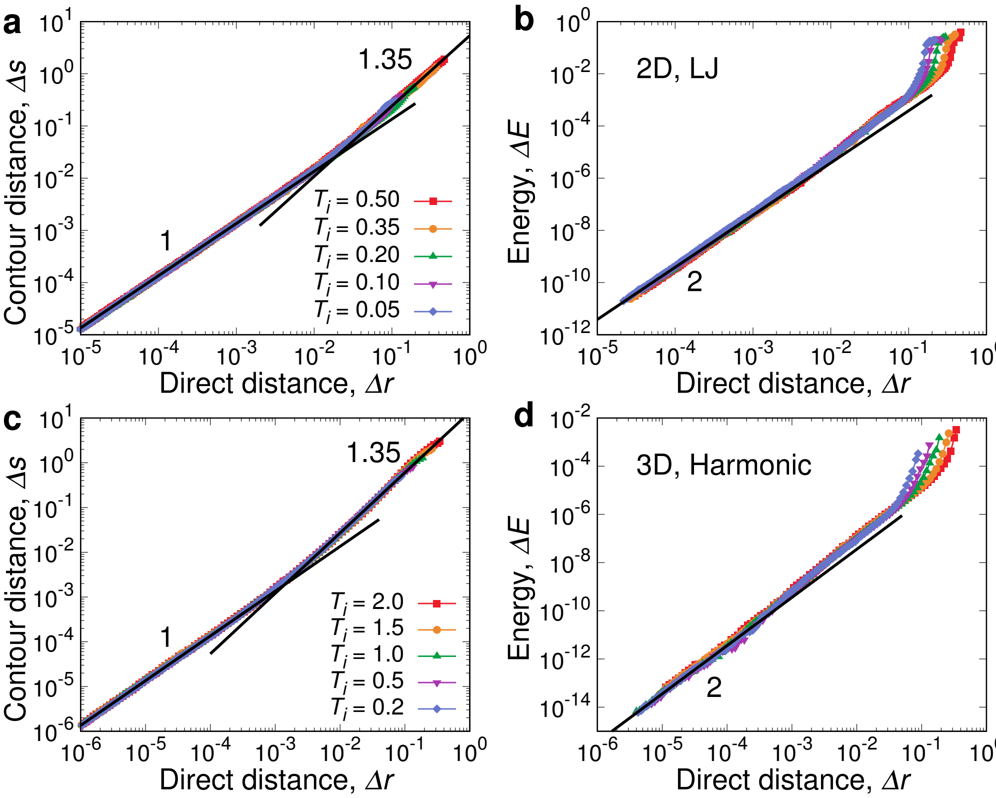

Supplementary Figure 12: **Fractal-like potential energy landscape in 2D Lennard-Jones and 3D harmonic systems.** Corresponding to Figs. 2b and 2c in the main text, finite-temperature configurations are relaxed to the inherent states (IS) of potential energy landscape (PEL) using energy minimisation (EM) methods. Top panels are for 2D Lennard-Jones systems. **a,** Relationship between contour distance Δs and direct distance Δr with respect to IS during EM for different initial temperatures T_i. Power-law fits are labelled by their corresponding exponent. A crossover from a linear relation $\Delta s \sim \Delta r$ close to IS to a fractal one $\Delta s \sim \Delta r^{1.35}$ away from IS is observed. **b,** Relationship between energy ΔE with respect to IS and Δr. The solid black lines indicate the quadratic (harmonic) relation $\Delta E \sim \Delta r^2$. A strong deviation from the harmonic approximation is observed at approximately the same Δr where the crossover into a fractal-like PEL happens. Bottom: **c,d,** The same analyses as in **a** and **b** for 3D harmonic systems. A crossover from a linear relation $\Delta s \sim \Delta r$ close to IS to a fractal one $\Delta s \sim \Delta r^{1.35}$ away from IS, and correspondingly a strong deviation from harmonic approximation is observed. Taken together, these results suggest a fractal potential energy landscape with a universal fractal dimension $d_{\mathrm{PEL}} = 1.35$ in different disordered systems. We emphasise that the thermal system (initial point of each curve in the top-right end) in physically relevant temperatures are completely out of the harmonic regime.

harmonic systems (see Fig. 1c in the main text). This result strongly suggests that the long-range stress correlation is a generic feature emerging when the system falls out of equilibrium and becomes trapped in the metastable glass state, which is independent of the interacting potential.

4. The fractal-like potential energy landscape in 2D Lennard-Jones and 3D harmonic systems. In Fig. 2 of the main text, we show that the potential energy landscape (PEL) of a 2D harmonic system has a fractal geometry, with a nontrivial fractal dimension $d_{\mathrm{PEL}} = 1.35$. Here we confirm that such a fractal-like PEL also exists in 2D Lennard-Jones and 3D harmonic systems. Most strikingly, as shown in Figs. 12a and 12c, the fractal dimension of PEL in both systems are found to be approximately $d_{\mathrm{PEL}} = 1.35$, the same as the 2D harmonic system. This result strongly suggests a universal fractal character of PEL, which represents an important aspect of the peculiar nature of amorphous solids. Moreover, a strong deviation from the harmonic energy expansion is found to coincide with the crossover into the fractal-like PEL, as shown in Figs. 12b and 12d. In conclusion, the general existence of giant anharmonicity controlled by the fractal-like PEL definitively rules out the effectiveness of harmonic approximations to understand the thermal amorphous solids from their inherent states.

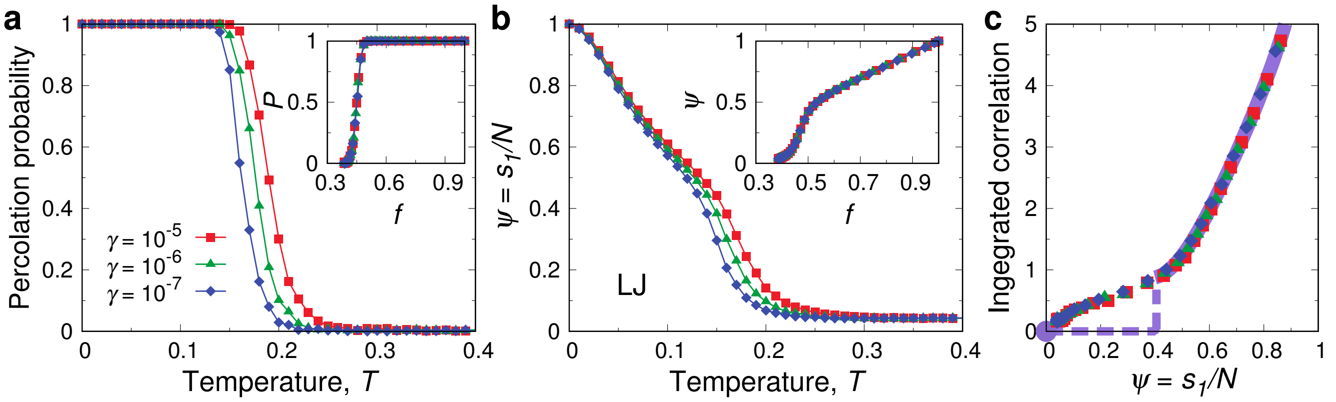

Supplementary Figure 13: **Percolation of force-bearing particles gives rise to long-range stress correlation in 2D Lennard-Jones systems. a,** Temperature dependence of percolation probability P for different γ. The γ-dependent non-equilibrium glass transition is signalled by the percolation of force-bearing particles. Inset: Collapse of P from different γ when replotted as a function of the fraction of force-bearing particles f. **b,** Temperature dependence of the relative size of the largest force-bearing cluster $\psi = s_1/N$ for different γ, with s_1 being the number of particles involved in the largest cluster. A strong similarity can be seen in comparison with the integrated stress-stress correlation in Fig. 11. Inset: Collapse of ψ from different γ when replotted as a function of f. **c,** Integrated shear-stress correlations for different cooling rates γ are plotted as a function of the relative size of the largest force-bearing network $\psi = s_1/N$. The filled circle and lines in purple have the same meaning as those in Fig. 4a of the main text. The nice collapse of the data at different γ suggests that the shear-stress correlation, more specifically, its far-field behaviour, is uniquely controlled by the largest force-bearing network. This result points to the general mechanism that the percolation of force-bearing particles gives rise to long-range stress correlation, irrespective of the interaction potential.

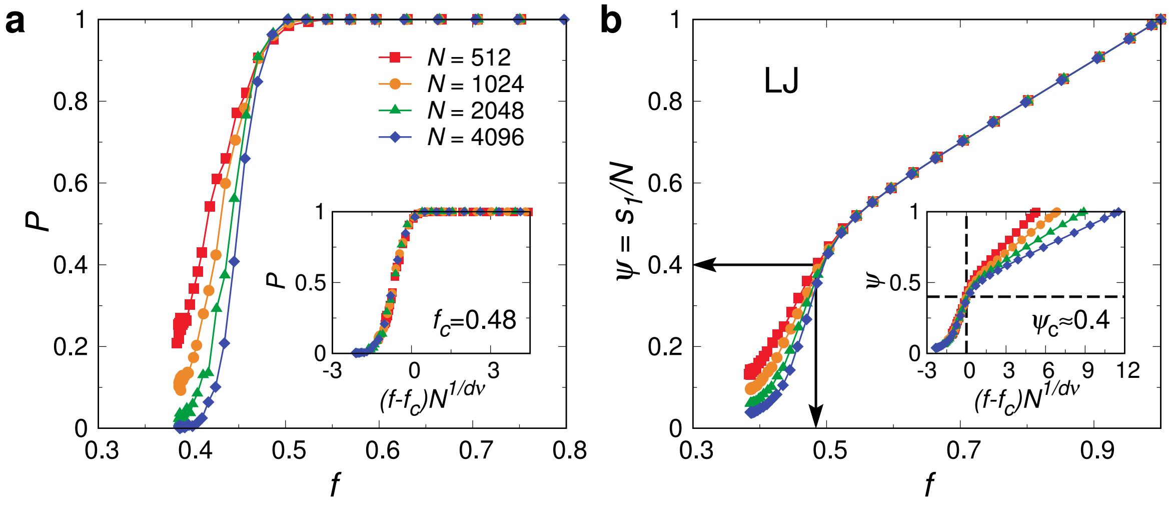

Supplementary Figure 14: **Finite-size scaling analysis for the percolation of force-bearing particles in 2D Lennard-Jones systems. a,** Percolation probability P as functions of the fraction of force-bearing particles f for different system sizes N at $\gamma = 10^{-6}$. Inset: Scaling collapse of P from different system sizes. Here $d = 2$ is the spatial dimension, $\nu = 4/3$ is the scaling exponent, and $f_c = 0.48$ is the critical occupation fraction. **b,** Corresponding to panel **a**, the relative size of the largest cluster $\psi = s_1/N$ as functions of f for different system sizes. The vertical and horizontal arrows indicate $f_c = 0.48$ and the corresponding ψ_c, respectively. Inset: Scaling collapse of ψ from different system sizes below the percolation transition, using the same f_c and ν as the inset of **a**. The horizontal dashed line indicates $\psi_c \approx 0.4$ at the percolation threshold f_c. Taken together, both panels **a** and **b** indicate that, when $N \to \infty$, an abrupt formation of system-spanning force-bearing network takes place across the non-equilibrium glass transition.

5. Percolation analysis of the force-bearing network in 2D Lennard-Jones systems. Here, we explore a possible generalisation of our force network analysis in 2D Lennard-Jones systems. Due to the long-range attractive

tail in the interaction, each particle may interact with many neighbours, making the force network highly complicated. However, it was shown through a study of boson peak in Lennard-Jones systems that a relatively few strong repulsive interactions (stiff contacts) play a dominate role, whereas the more numerous weaker interactions can be treated as perturbations [29]. This means that, although intrinsically complex, the identification of strong backbone may capture the main effects of the force network even in Lennard-Jones systems. Our definition of the force-bearing network is indeed based on the same physical consideration. However, we find that it is necessary to extend the criterion to $F_{\rm str} < 1.28$ to properly identify the force-bearing particles, which may be due to the renormalising effect of long-range attractions. Figure 13a shows the temperature dependence of the percolation probability for three different cooling rates. It is seen that the percolation of force-bearing particles takes place at a γ-dependent glass transition temperature, in good agreement with the emergence of the long-range stress correlation shown in Fig. 11. In addition, the temperature dependence of the relative size of the largest force-bearing cluster $\psi = s_1/N$ is shown in Fig. 13b, where a close similarity compared with Fig. 11 can be observed. In Fig. 13c, We further plot the integrated shear-stress correlations as a function of the relative size of the largest force-bearing network ψ and find a nice collapse of the data from different cooling rates. In conclusion, these results suggest that the long-range stress correlation in LJ systems is also uniquely controlled by the underlying percolating force-bearing network, which therefore points to a general understanding of the non-equilibrium glass transition from the mechanical perspective.

Finite-size scaling analysis for the percolation of force-bearing particles is carried out to support the plot of Fig. 13. As shown in Fig. 14a, the transition of percolation probability P from 0 to 1 as a function of the fraction of force-bearing particles f becomes steeper with increasing the system size N. Scaling collapse of P is realised in the inset of Fig. 14a, indicating a well-defined percolation transition at $f_c = 0.48$ in the large system size limit. Correspondingly, the relative size of the largest force-bearing network ψ as a function of f is shown in Fig. 14b, with the finite-size scaling collapse of ψ below f_c given in the inset. This result again indicates that a system-spanning force-bearing network with $\psi \approx 0.4$ emerges abruptly at f_c as $N \to \infty$. Therefore, a well-defined percolation transition of the force-bearing network in the large system size limit is also expected in LJ systems. Taken together results from both harmonic and LJ systems, the percolation of the force-bearing network that gives rise to the long-range stress correlation is speculated to be the universal physical mechanism underlying the non-equilibrium glass transition.

SUPPLEMENTARY REFERENCES

[1] Allen, M. P. & Tildesley, D. J. *Computer simulation of liquids* (Oxford university press, 2017).

[2] Mazoyer, S., Ebert, F., Maret, G. & Keim, P. Dynamics of particles and cages in an experimental 2D glass former. *Europhys. Lett.* **88**, 66004 (2010).

[3] Vivek, S., Kelleher, C. P., Chaikin, P. M. & Weeks, E. R. Long-wavelength fluctuations and the glass transition in two dimensions and three dimensions. *Proc. Natl Acad. Sci. USA* **114**, 1850–1855 (2017).

[4] Flenner, E. & Szamel, G. Fundamental differences between glassy dynamics in two and three dimensions. *Nat. Commun.* **6**, 7392 (2015).

[5] Shiba, H., Yamada, Y., Kawasaki, T. & Kim, K. Unveiling dimensionality dependence of glassy dynamics: 2D infinite fluctuation eclipses inherent structural relaxation. *Phys. Rev. Lett.* **117**, 245701 (2016).

[6] Illing, B. *et al.* Mermin–wagner fluctuations in 2D amorphous solids. *Proc. Natl Acad. Sci. USA* **114**, 1856–1861 (2017).

[7] Greer, A. L. New horizons for glass formation and stability. *Nat. Mater.* **14**, 542 (2015).

[8] Yanagishima, T., Russo, J. & Tanaka, H. Common mechanism of thermodynamic and mechanical origin for ageing and crystallization of glasses. *Nat. Commun.* **8**, 15954 (2017).

[9] Struik, L. C. E. *Physical aging in amorphous polymers and other materials* (Elsevier, Amsterdam, 1978).

[10] Stauffer, D. & Aharony, A. *Introduction to percolation theory* (Taylor & Francis, 1994).

[11] Schrenk, K. J. *et al.* Percolation with long-range correlated disorder. *Phys. Rev. E* **88**, 052102 (2013).

[12] Sheinman, M., Sharma, A., Alvarado, J., Koenderink, G. & MacKintosh, F. Anomalous discontinuity at the percolation critical point of active gels. *Phys. Rev. Lett.* **114**, 098104 (2015).

[13] Alvarado, J., Sheinman, M., Sharma, A., MacKintosh, F. C. & Koenderink, G. H. Molecular motors robustly drive active gels to a critically connected state. *Nat. Phys.* **9**, 591 (2013).

[14] Hu, H., Ziff, R. M. & Deng, Y. No-enclave percolation corresponds to holes in the cluster backbone. *Phys. Rev. Lett.* **117**, 185701 (2016).

[15] Achlioptas, D., D'souza, R. M. & Spencer, J. Explosive percolation in random networks. *Science* **323**, 1453–1455 (2009).

[16] Friedman, E. J. & Landsberg, A. S. Construction and analysis of random networks with explosive percolation. *Phys. Rev. Lett.* **103**, 255701 (2009).

[17] da Costa, R. A., Dorogovtsev, S. N., Goltsev, A. V. & Mendes, J. F. F. Explosive percolation transition is actually continuous. *Phys. Rev. Lett.* **105**, 255701 (2010).

[18] Riordan, O. & Warnke, L. Explosive percolation is continuous. *Science* **333**, 322–324 (2011).

[19] Cho, Y. S., Hwang, S., Herrmann, H. J. & Kahng, B. Avoiding a spanning cluster in percolation models. *Science* **339**, 1185–1187 (2013).

[20] Yang, X., Tong, H., Wang, W.-H. & Chen, K. Emergence and percolation of rigid domains during the colloidal glass transition. *Phys. Rev. E* **99**, 062610 (2019).

[21] Mishra, C. K., Ma, X., Habdas, P., Aptowicz, K. B. & Yodh, A. G. Correlations between short-and long-time relaxation in colloidal supercooled liquids and glasses. *Phys. Rev. E* **100**, 020603 (2019).

[22] Liu, A. J. & Nagel, S. R. The jamming transition and the marginally jammed solid. *Annu. Rev. Condens. Matter Phys.* **1**, 347–369 (2010).

[23] Tong, H. & Tanaka, H. Revealing hidden structural order controlling both fast and slow glassy dynamics in supercooled liquids. *Phys. Rev. X* **8**, 011041 (2018).

[24] Kob, W. & Andersen, H. C. Testing mode-coupling theory for a supercooled binary lennard-jones mixture i: The van hove correlation function. *Phys. Rev. E* **51**, 4626 (1995).

[25] Kobayashi, S., Maeda, K. & Takeuchi, S. Computer simulation of deformation of amorphous cu57zr43. *Acta Metall.* **28**, 1641–1652 (1980).

[26] Berthier, L., Biroli, G., Bouchaud, J.-P., Cipelletti, L. & van Saarloos, W. (eds.) *Dynamical Heterogeneities in Glasses, Colloids, and Granular Media*, vol. 150 (Oxford University Press, Oxford, 2011).

[27] Schuh, C. A. & Lund, A. C. Atomistic basis for the plastic yield criterion of metallic glass. *Nat. Mater.* **2**, 449 (2003).

[28] Cao, P., Short, M. P. & Yip, S. Potential energy landscape activations governing plastic flows in glass rheology. *Proc. Natl Acad. Sci. USA* **116**, 1879018797 (2019).

[29] Xu, N., Wyart, M., Liu, A. J. & Nagel, S. R. Excess vibrational modes and the boson peak in model glasses. *Phys. Rev. Lett.* **98**, 175502 (2007).
